# Discovery of Small Molecule NSC290956 as a Therapeutic Agent for KRas Mutant Non-Small-Cell Lung Cancer

**DOI:** 10.3389/fphar.2021.797821

**Published:** 2022-01-05

**Authors:** Jiaxin Zhang, Zuojia Liu, Wenjing Zhao, Xunzhe Yin, Xiliang Zheng, Chuanbo Liu, Jin Wang, Erkang Wang

**Affiliations:** ^1^ State Key Laboratory of Electroanalytical Chemistry, Changchun Institute of Applied Chemistry, Chinese Academy of Sciences, Changchun, China; ^2^ Department of Chemistry, University of Science and Technology of China, Hefei, China; ^3^ Department of Chemistry and Physics, State University of New York, Stony Brook, NY, United States

**Keywords:** specificity-affinity screening, cancer metabolism, drug discovery, non-small-cell lung cancer, open conformation of Ras

## Abstract

HRas-GTP has a transient intermediate state with a “non-signaling open conformation” in GTP hydrolysis and nucleotide exchange. Due to the same hydrolysis process and the structural homology, it can be speculated that the active KRas adopts the same characteristics with the “open conformation.” This implies that agents locking this “open conformation” may theoretically block KRas-dependent signaling. Applying our specificity-affinity drug screening approach, NSC290956 was chosen by high affinity and specificity interaction with the “open conformation” structure HRasG60A-GppNp. In mutant KRas-driven non-small-cell lung cancer (NSCLC) model system, NSC290956 effectively suppresses the KRas-GTP state and gives pharmacological KRas inhibition with concomitant blockages of both the MAPK-ERK and AKT-mTOR pathways. The dual inhibitory effects lead to the metabolic phenotype switching from glycolysis to mitochondrial metabolism, which promotes the cancer cell death. In the xenograft model, NSC290956 significantly reduces H358 tumor growth in nude mice by mechanisms similar to those observed in the cells. Our work indicates that NSC290956 can be a promising agent for the mutant KRas-driven NSCLC therapy.

## Introduction

Ras proteins function in many signaling pathways as a binary molecular switch cycling between an active guanosine triphosphate (GTP)-bound (RasT) and an inactive guanosine diphosphate (GDP)-bound (RasD) state (Pylayeva-Gupta et al., 2011; Hobbs et al., 2016). In tumor, Ras is stuck in a permanently active RasT state (Burns et al., 2014). At this form, RasT continuously activates diverse down-stream effectors. Subsequently, the activated state is terminated by converting RasT to RasD state. Proper regulation of Ras cycling is essential for cellular functions; thus, the dysregulation is a hallmark of tumor (Hanahan and Weinberg, 2011).

GTP hydrolysis is in equilibrium with three distinct but equally populated conformations, one of which is the “non-signaling open conformation” (Ford et al., 2005). Nassar et al. revealed that HRasG60A-GppNp complex was found to adopt an “open conformation” at the switch 1 region and to abolish the biological activity of HRas (Hwang et al., 1996; Ford et al., 2005). Initially these results were obtained by using HRas proteins, but the states also occur in KRas (Parker et al., 1993). In cancer cells the mutant KRas accumulates in an elevated GTP-bound proportion and thus leads to a constantly activated form. Once at the KRasT state, this reaction of GTP hydrolysis and nucleotide exchange occurs immediately. Insights into KRas dynamic from experimental data showed that specific mutations in the switches (D33E in switch 1 or A59G in switch 2) (Lu et al., 2018), replacing Y32 in switch 1 with other amino acids (Spoerner et al., 2010), and phosphorylating tyrosine residues Y32 in switch 1 and Y64 in switch 2 induced the equilibrium towards an “open conformation” (Kano et al., 2019). Recent crystal structures combined with molecular dynamic simulations indicated extremely open switch 1 conformations of KRas (Pantsar, 2020). This implies that the “open conformation” may be a convergent point for survival signaling in KRas-driven cancer.

Given frequent addiction to oncogenic KRas, targeting the transition state structure would be therapeutically beneficial. (1) Targeting a transition state structure by drug design was known as an effective approach for inhibiting an enzymatic reaction. (2) Global genomic analysis revealed that drugs targeting key nodal points could be preferable for therapies. In principle, a drug directed at targeting this intermediate transition conformation might act as a “gatekeeper” that keeps KRas in a conformation incompatible with downstream effectors binding through decreasing the proportion of KRasT state (Sun et al., 2012). Along this line, such a drug ultimately has a pharmacological inhibition of KRas oncoproteins, thereby interrupting KRas-dependent survival in the cell.

**FIGURE 1:**
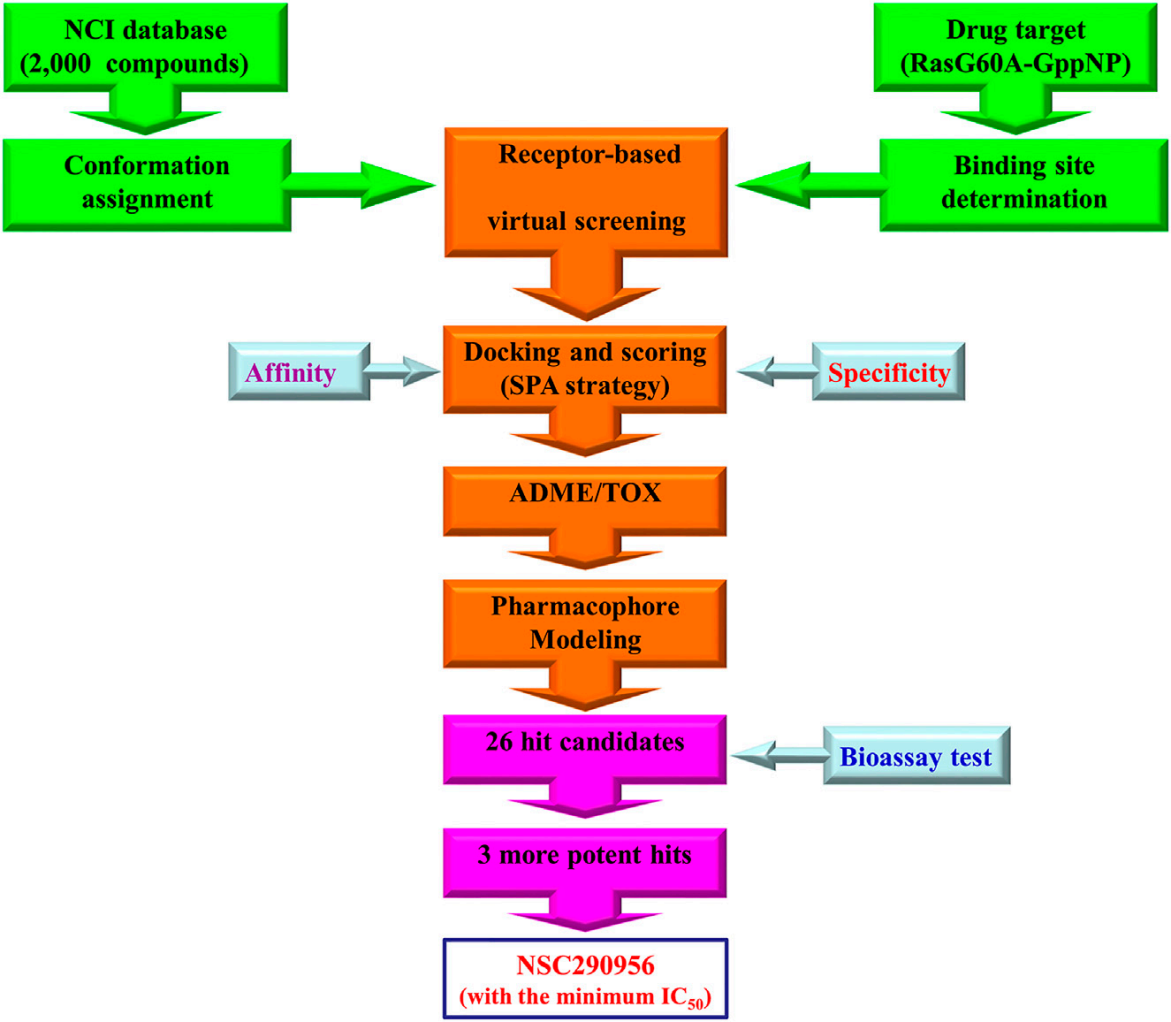
Workflow of specificity-affinity virtual screening strategy. Out of this screening, 26 small-molecule compounds were selected for further bioactivity testing with three hits discovered. Thereafter, NSC290956 was picked out due to its minimum IC_50_ value.

In this study, we take advantage of the finding to develop small molecule inhibitors that stabilize the “open conformation” of activated KRas in tumor cell, thus preventing KRas from converting to its signaling one. Given the nearly 25% frequency of KRas mutations in non-small-cell lung cancer (NSCLC), we set out to utilize NSCLC as a model system to identify this novel antitumor strategy for targeting the intermediate conformation structure. We use a multidisciplinary approach based on the synergy of computational, biophysical, and biological methods to identify the “open conformation” as a potential target. Applying a specificity-affinity virtual screening workflow (Figure 1), we discover a small molecule NSC290956. Combining data from ensemble docking *in silico*, binding interactions *in vitro* and *in vivo*, and experiments in intact cells and mouse models, we show that NSC290956 specifically binds to the “open conformation” structure, effectively attenuates KRas functions, and significantly reduces NSCLC cell survival and xenograft tumor growth in mice. This work provides a functional insight into the “open conformation” and validates a promising therapeutic strategy for KRas-dependent cancer.

## Results

### Virtual Screening and Hit Validation

**FIGURE 2:**
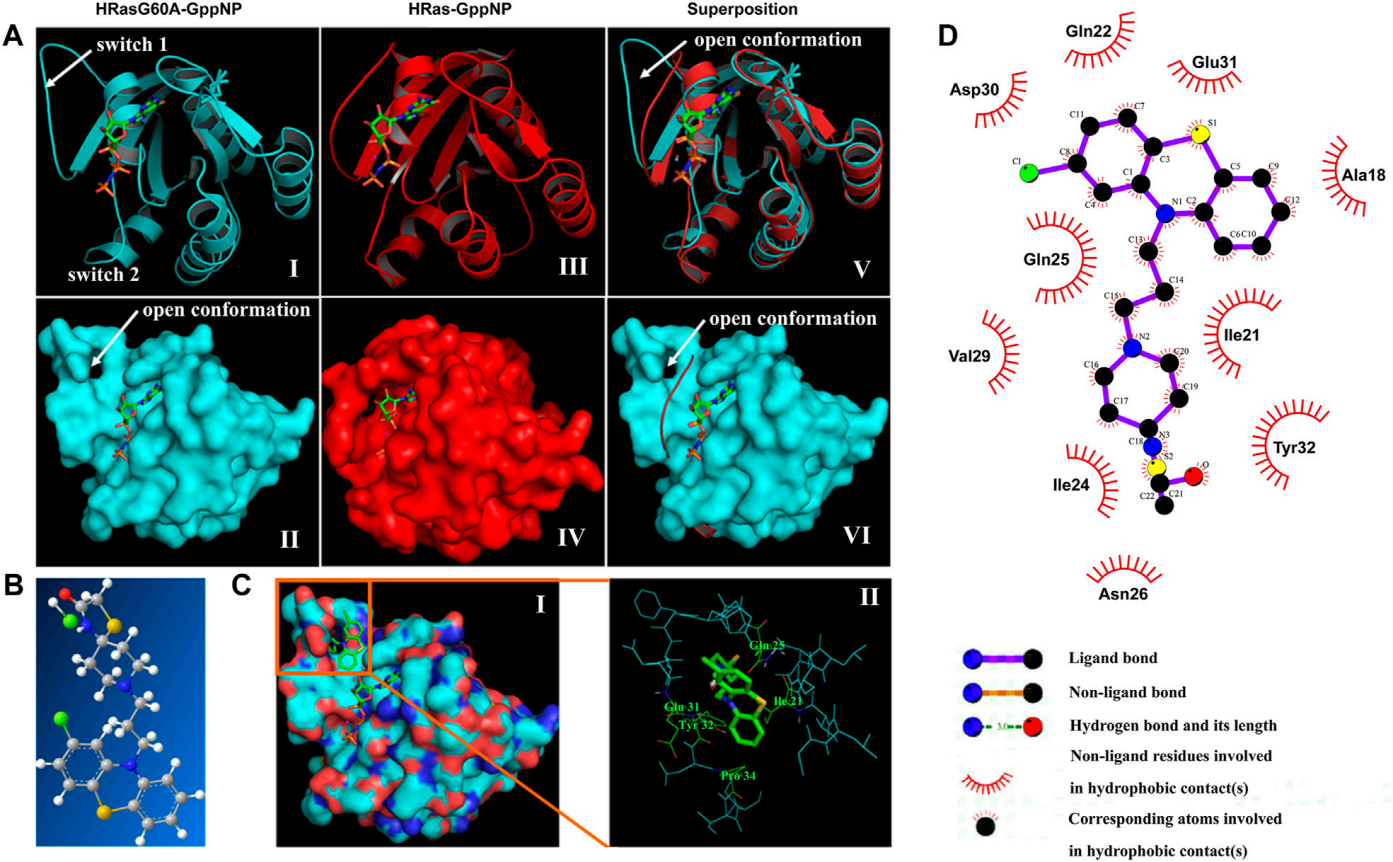
*In silico* virtual screening identifies NSC290956 as a hit compound. **(A)** Structure models of HRasG60A-GppNp (PDB ID: 1XCM), HRas-GppNp (PDB ID: 2RGE), and superposition of the two structures as a ribbon **(top)** and surface **(bottom)** representations. The switch regions (switch 1 & 2) are indicated. GppNp is shown as a stick model. Mg^2+^ is shown as a cyan sphere in HRasG60A-GppNp or red sphere in HRas-GppNp, respectively. **(B)** Chemical structure of NSC290956 (C, grey; O, red; N, blue; H, white; S, yellow; and Cl, green). **(C)** Surface representation shows NSC290956 binding to the cavity on HRasG60A-GppNp. GppNp and NSC290956 are shown as stick models **(i)**. Residues involving intermolecular interactions and NSC290956 (green) are shown on the backbone structure of binding cavity of HRasG60A-GppNp **(ii)**. **(D)** Schematic diagram shows putative interaction between NSC290956 and HRasG60A-GppNp. Compound is shown in purple stick (C, black; O, red; N, blue; S, yellow; and Cl, green). Residues involving hydrophobic interactions are shown as starbursts.

Comparing the conformation difference between GppNp-bound HRasG60A (PDB: 1XCM) and HRas (PDB: 2RGE), an unexpected characteristic of HRasG60A-GppNp structure is the remodeling of the dynamic at the switch 1 region to a conformation that completely pulls it away from nucleotide into solvent (Figures 2Ai,iii). And the dynamic at switch 2 region is restructured and moves away from nucleotide (Ford et al., 2005). Superposition shows that mutating Gly60 to Ala leads to a dramatic opening of switch 1 (images v and vi). This means that an “open conformation” is formed on the surface of HRasG60A-GppNp. Structural analysis further shows that the “open conformation” is remarkably similar to the nucleotide free HRas (NF-HRas) as found in the HRas/Sos complex (Boriack-Sjodin et al., 1998), which constitutes an intermediate for nucleotide exchange (Lenzen et al., 1998). Accordingly, HRasG60A-GppNp has the same characteristic with the transient intermediate for nucleotide exchange right after GTP binding to NF-HRas but before the final GTP-bound conformation was reached (Ford et al., 2005). Consequently, the “open conformation” possesses a dominant negative characteristic, losing its ability to activate downstream effectors in the cells (Sung et al., 1996; Ford et al., 2005).

The surface cavity as an “open conformation” (images ii and iv) to be frozen appears as a target suitable for drug binding. We used a two-dimension screening strategy to dock compounds to this binding cavity individually. This process led to the selection of 26 compounds by scoring the binding energies and calculating the intrinsic specificity ratios (ISR) (Supplementary Tables S1, S2). After testing these candidates, we focused on the most promising compound NSC290956 (Figure 2B; Supplementary Figure S1A). In *in silico* docking model, NSC290956 was trapped in the binding cavity between the switch 1 region and the relevant residues (Figure 2C).

The predicted interaction between NSC290956 and HRasG60A-GppNp was generated by the computational model (Figure 2D). This hydrophobic cavity is occupied by the aromatic phenothiazine group of NSC290956 with chloro substitute providing tight van der Waals contacts. The corresponding atoms contained in the phenothiazine group lead to hydrophobic interactions with residues Ala18, Gln22, Asp30, and Glu31. The diazaspiro decane group of the warhead of NSC290956 is inserted into the cavity pocket, leading to hydrophobic interactions with residues Ile21, Ile24, and Tyr32. Accordingly, an extensive hydrogen bonding network is formed between NSC290956 and the corresponding residues in the switch 1 region.

### Preparation and Identification of NSC290956 as a Potential Inhibitor of KRas

**FIGURE 3:**
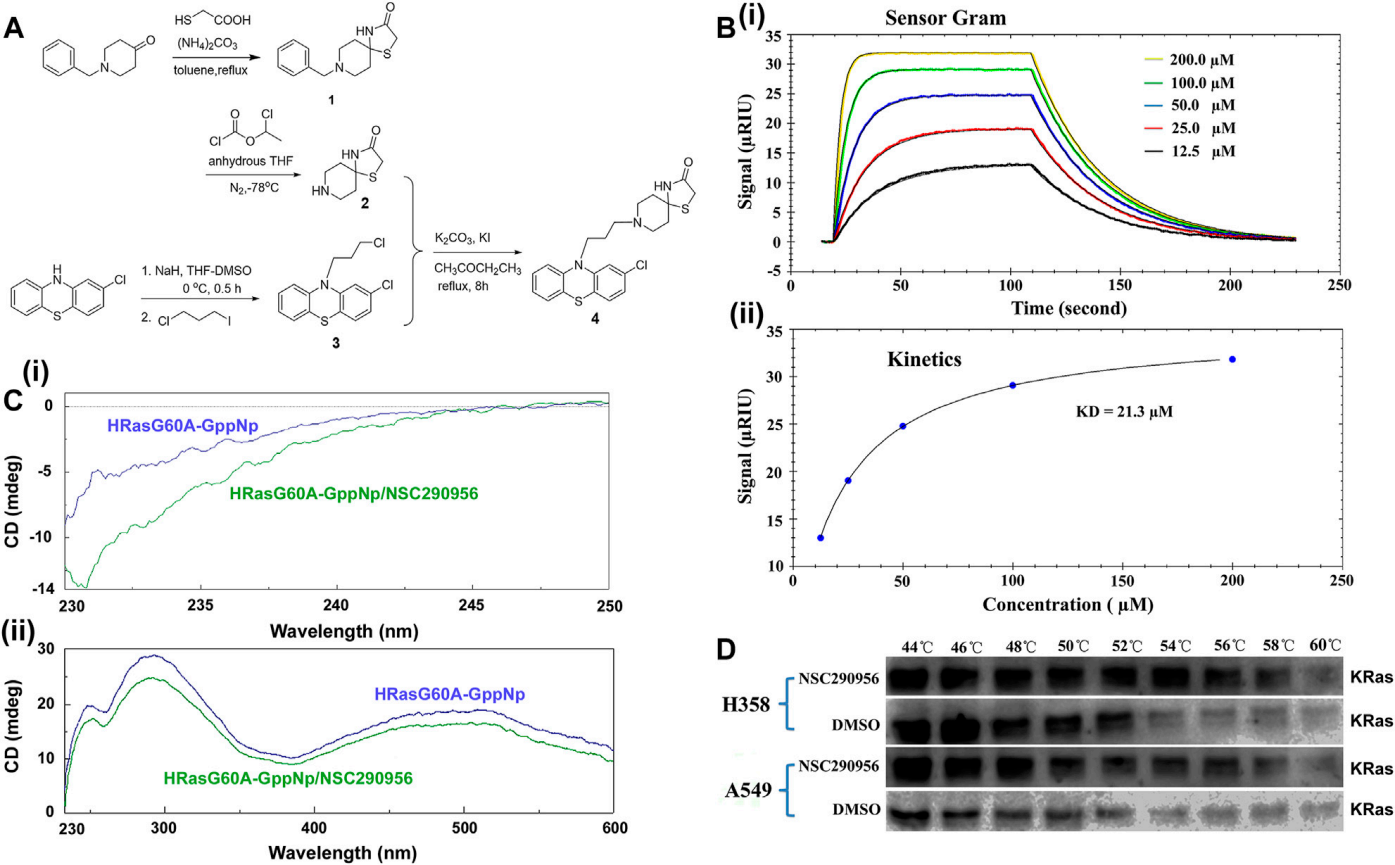
Preparation and identification of NSC290956 as a potential inhibitor of KRas. **(A)** The synthetic route of NSC290956. **(B)** Representative SPR assay confirmed the interaction between NSC290956 and HRasG60A-GppNp. The response signal (RIU) as a function of time was measured with increasing concentrations of NSC290956 **(i)**. Graph of equilibrium RIU responses versus compound concentrations was plotted **(ii)**. **(C)** Circular dichroism spectra displayed the secondary **(i)** and the tertiary **(ii)** structure of HRasG60A-GppNp in the presence of NSC290956, respectively. **(D)** Representative western blots were shown for the stabilization of KRas.

The hit candidate NSC290956, chemical name 1-Thia-4,8-diazaspiro[4.5]decan-3- one,8-[3-(2-chloro-10H-phenothiazin-10-yl)propyl], was prepared by following the synthetic route illustrated in the scheme in Figure 3A and confirmed by ESI-MS spectra (Supplementary Figure S1B). The detailed synthetic procedures and characterizations of intermediate compounds, including the original NMR and ESI-MS spectra, are described in the Supplementary Material. Subsequently, the interaction between NSC290956 and the HRasG60A-GppNp complex was experimentally identified by the surface plasmon resonance (SPR) assay. The binding curves of association/dissociation and the steady-state analysis disclosed a high affinity with KD value of 21.3 μM (Figure 3B). In contrast, HRas-GppNp did not appear to interact with NSC290956 in the identical conditions (Supplementary Figure S2). This observation clearly demonstrates that the specific binding pocket only exists in HRasG60A-GppNp. In addition, circular dichroism spectroscopy uncovered that the secondary and the tertiary structure of HRasG60A-GppNp was respectively disrupted with the addition of NSC290956 (Figure 3C), which may be a strong identification for the specific interaction.

Considering the nearly 25% frequency of KRas mutations in non-small-cell lung cancer (NSCLC), we set out to use diverse KRas-expressing NSCLC cell models to identify this novel antitumor strategy. In mutant KRas-driven cancer cells, the “open conformation” dominantly results from the activated KRas due to the “open conformation” accounting for more partition in mutated KRas than in wild-type HRas and NRas (Ford et al., 2005; Janes et al., 2018). Based on the target engagement principle (Martinez Molina et al., 2013), we performed the cellular thermal shift assay (CETSA) to verify the cellular uptake and target engagement of NSC290956 in KRas-addicted H358 (KRasG12C) and A549 (KRasG12S) cell lines. KRas alone (DMSO as control) denatured around 52–54°C within cells. The addition of NSC290956 (25 μM) stabilized KRas by 4–6°C (Figure 3D). The observations indicate that NSC290956 directly binds to and thermally stabilizes KRas to a certain extent with increasing the denaturation temperature. Combining the specific *in vitro* interaction between NSC290956 and the “open conformation” structure, the direct *in vivo* interaction supports the existence of the “open conformation” structure in the activated KRas cells as the cellular target of NSC290956 in the subsequent cell-based assays.

### NSC290956 Inhibited Cellular KRas Activity and Downstream Signaling Events

**FIGURE 4:**
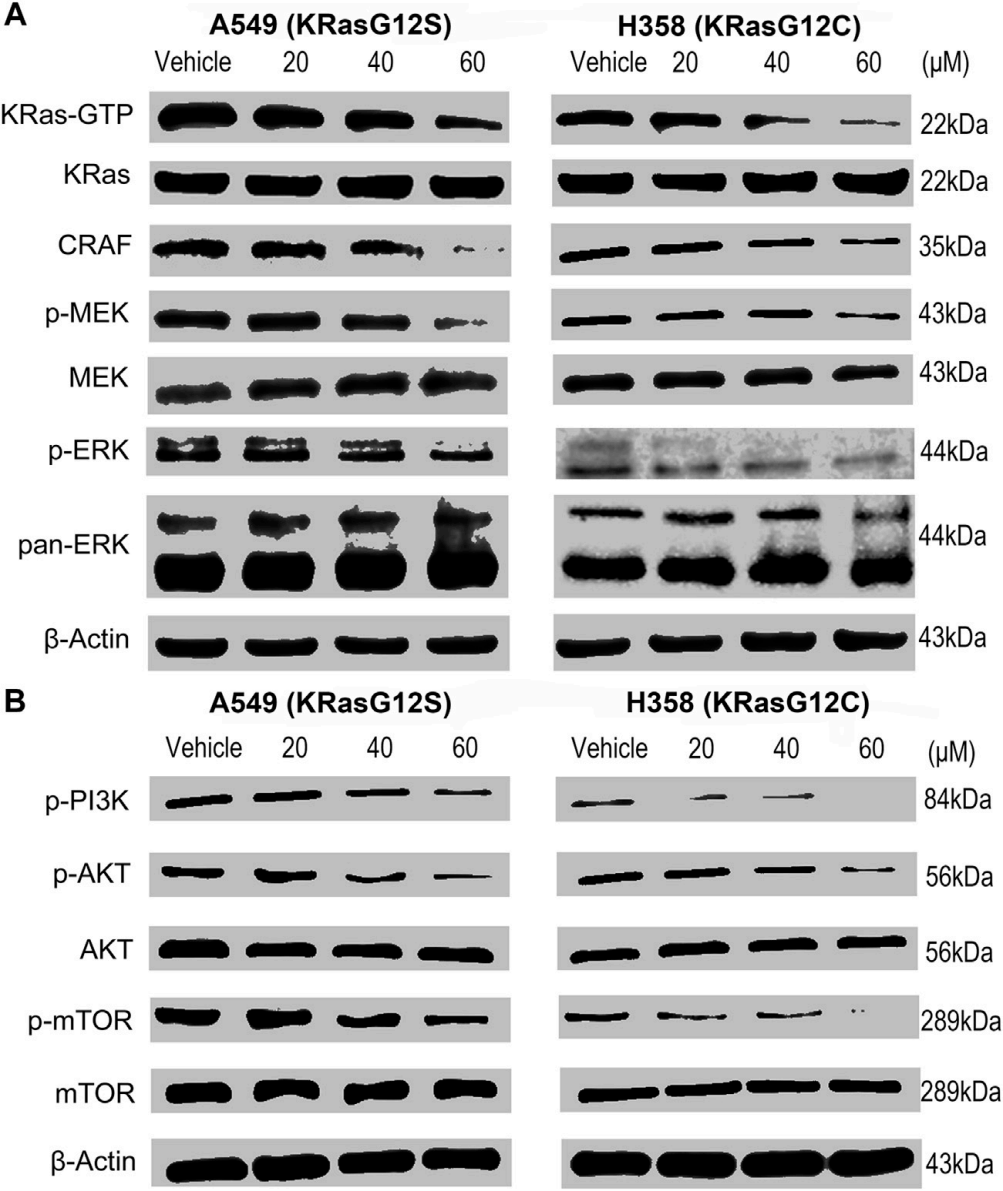
NSC290956 inhibites KRas activity and downstream signaling events in KRas-driven nuytant NSCLC cells. **(A)** Pull-down of active GTP-bound KRas by Raf-RBD after exposure to NSC290956 showed a comparative decrease in the KRas-GTP and the RAF-MEK-ERK signaling. **(B)** KRas inactivation inhibited the PI3K-AKT-mTOR signaling in both the cell lines. β-Actin was used as the internal control.

In theory, NSC290956 binding to the “open conformation” might keep KRas at an inactive state and thereafter result in the cellular KRas-GTP attenuation. To verify this prediction, we evaluated the effect of NSC290956 blocking KRas activation on mutant A549 and H358 cell lines. These cells were serum-starved in the cultures and stimulated with EGF (that activates the MAPK cascade) before harvest. Thereafter, the KRas-GTP level was determined using an active KRas pull-down assay. As expected, a decrease of KRas-GTP was clearly detected in both the cell lines in a concentration-dependent manner, but no effect was observed on total KRas (Figure 4A).

We further found that both A549 and H358 cell lines showed the decreased CRAF abundance and the reduced MEK and ERK1/2 phosphorylations after NSC290956 treatment. By comparison, no obvious changes in the total MEK and pan-ERK1/2 were detected in cells (Figure 4A). Simultaneously, the phosphorylations of PI3K, AKT, and mTOR were inhibited equally well (Figure 4B). In contrast, NSC290956 treatment did little to alter their total expressions when compared to control cells. The cell-based assays indicated that NSC290956 exerted a broad spectrum blockage of KRas downstream signaling events by direct blocking KRas activity (Supplementary Figure S3).

### NSC290956 Decreased the Viability, Cell Cycle, and Migration of Non-Small-Cell Lung Cancer Cells

**FIGURE 5:**
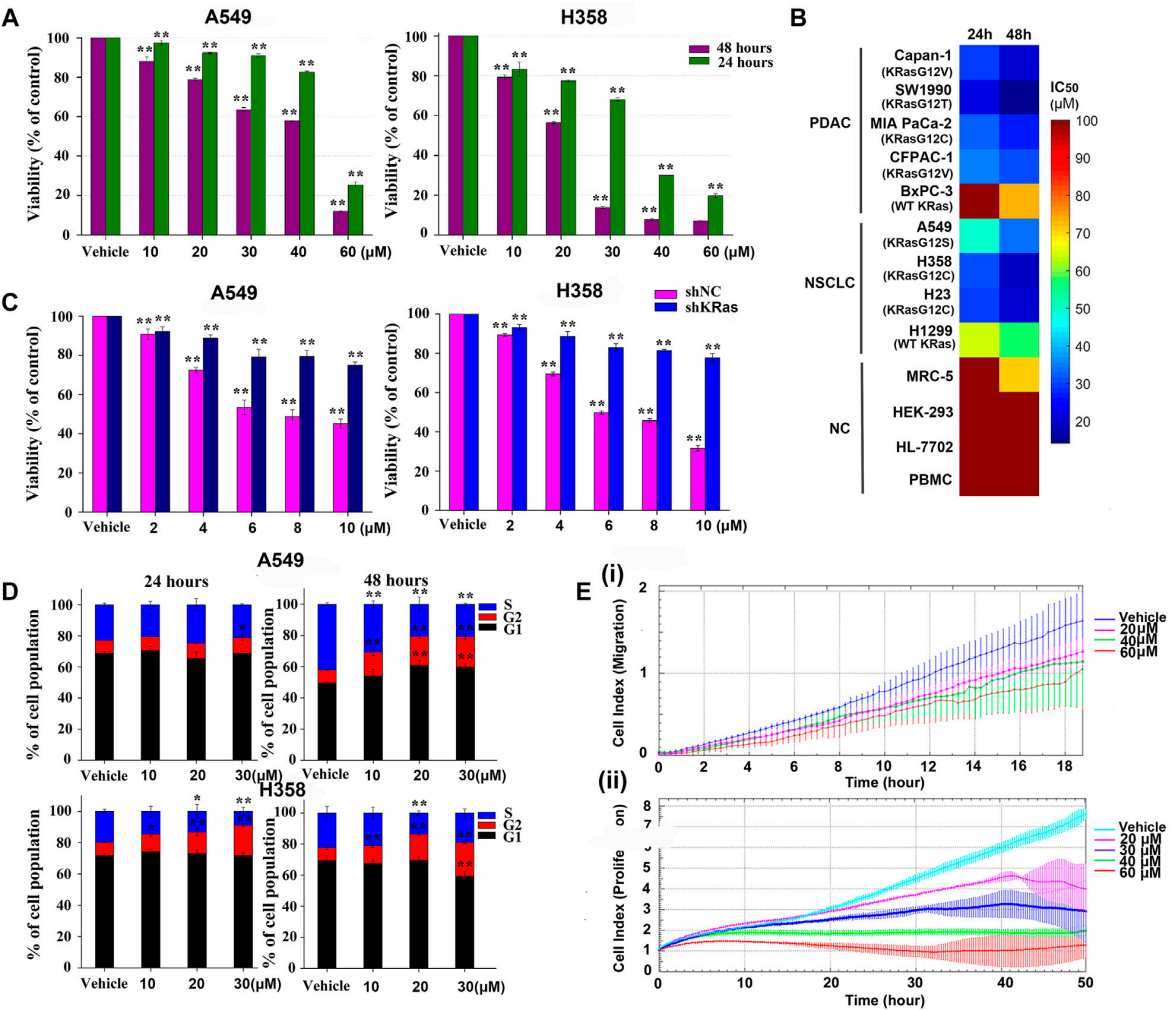
NSC290956 decreases the cellular viability, cell cycle, and migration. **(A)** The survival effect of NSC290956 on representative A549 and H358 cell lines for 24 and 48 h, respectively. **(B)** A broader inhibitory effect of NSC290956 on various KRas-driven human pancreatic cancer (PDAC) cell lines (KRasG12V Capan-1, KRasG12T SW1990, KRasG12C MIA PaCa-2, KRasG12V CFPAC-1, and WT KRas BxPC-3), NSCLC cell lines (KRasG12S A549, KRasG12C H358, KRasG12C H23, and WT KRas H1299), and normal cells (NC) such as (MRC-5, HEK-293, HL-7702, and PBMC) was examined by exposure to NSC290956. **(C)** Effect of KRas deletion on NSC290956-induced growth inhibition in colony formation assay. Effect of shNC or shRNA plus drug treatment on colony formation was shown as the percentage of DMSO-treated control. **(D)** Effect of NSC290956 on cell cycle progression in the two cancer cell lines treated for 24 and 48 h, respectively. **(E)** Real-time cell index analysis of A549 cell migration over 18 h **(i)** and cell proliferation over 50 h **(ii)**. The results were presented as the mean ± SD of triplicate experiments. Significant differences from untreated control were indicated *p < 0.05 and **p < 0.01.

To assess the effect of NSC290956 on KRas-dependent cell survival, both A549 and H358 cells were respectively exposed to a range of NSC290956 concentrations, resulting in reduced viability (Figure 5A). We further tested a broader panel of KRas-driven mutant (KRasG12V CFPAC-1, KRasG12C MIA PaCa-2, KRasG12V Capan-1, KRasG12T SW1990 and KRasG12C H23) and wild-type KRas (BxPC-3 and H1299) cancer cells as well as human normal peripheral blood mononuclear cell (PBMC), embryonic kidney (HEK-293), lung fibroblast (MRC-5), and liver (HL-7702) cell lines. The dramatically reduced survival was observed in mutant KRas-driven cancer cells, while no significant growth inhibition was observed for the normal cells (Figure 5B). These observations raise the intriguing possibility that although all KRas mutations stabilize KRas-GTP levels, the different KRas oncogenic alleles may have distinct intrinsic hydrolysis rates of GTP.

To rule out the off-target effect, lentiviral short-hairpin RNA (shRNA) was introduced to suppress the expression of KRas in both cancer cell lines. The number of KRas in the shKRas cells was much less than that in the control or the negative control (shNC) cells (Supplementary Figures S4A,B). It’s worth noting that both cell lines were sensitive to KRas knockdown (Supplementary Figure S4C). The effect of shRNA alone or plus drug treatment on colony formation was shown as the percentage of DMSO-treated control (Figure 5C). Knockdown of KRas from these cells resulted in resistance to NSC290956, indicating a degree of KRas-dependent lethality. These findings support an on-target mechanism of action and a KRas-mutation specific way of function within the cells.

Simultaneously, the impact of NSC290956 on cell cycle related to cell growth was examined in A549 and H358 cell lines. A time- and concentration-dependent increase in the cell population at G2/M phase was observed intuitively (Figure 5D). CDK1 and Cyclin B1 expressions involving in G2/M phase displayed the significantly decreased levels in the two cell lines (Supplementary Figure S5). It can be concluded that NSC290956 resulted in growth inhibition that elicited a prominent, prolonged accumulation of cells at G2/M phase in the cells. As an example, the real-time xCELLigence system analysis demonstrated that NSC290956-treated A549 cells showed the reduced migration in addition to the suppressed proliferation (Figure 5E). Of note, when the concentration was increased to 60 µM, cell growth was near complete abrogation in these cases. These results suggest that NSC290956 rather effectively suppresses the survival and proliferation of KRasG12C-driven H358 cells dependent phenotypes.

### NSC290956 Changed KRas-Dependent Metabolic Phenotype in Non-Small-Cell Lung Cancer Cells

**FIGURE 6:**
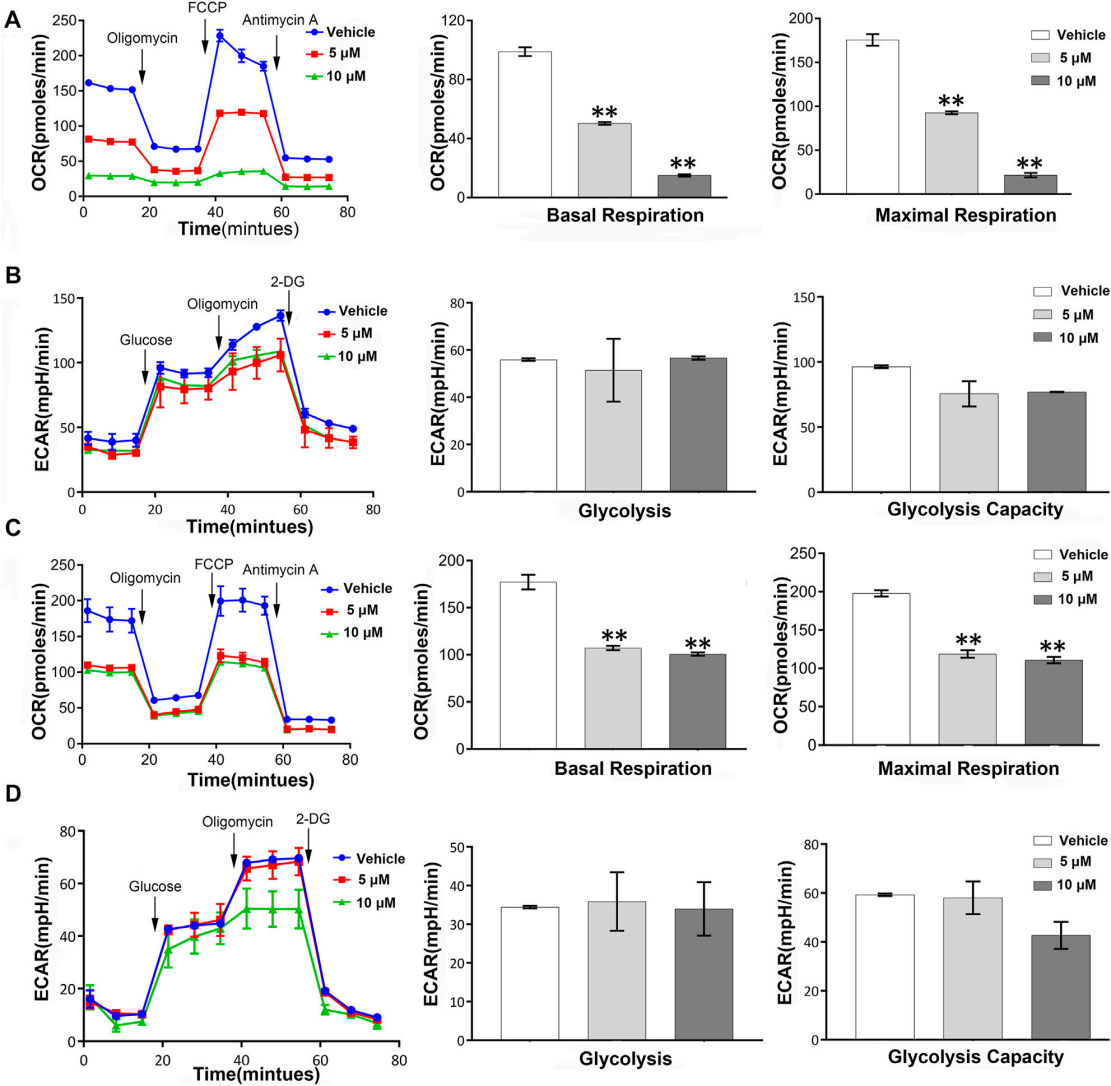
Mitochondrial metabolism is required for KRas-mediated energy switching in both the cell lines. Representative basal OCR and ECAR values were measured in A549 cells **(A,B)** and H358 cells **(C,D)** in the presence of NSC290956. Seahorse assays were performed by using an XFp Extracellular Flux Analyzer. Cells were treated for 24 h then sequentially injected with 1 μM oligomycin, 0.5 μM FCCP, and 0.5 μM anti-mycin A/0.5 μM rotenone for OCR test or with glucose, oligomycin, and 2-DG for ECAR test. Histogram data are the mean ± SD of three replicates.

Metabolic regulation is essential for the maintenance of proliferation in tumor cell. We therefore questioned whether the anti-proliferation of NSC290956 is associated with metabolic phenotype switching between mitochondrial respiration and glycolysis in H358 and A549 cells. Applying Seahorse testing, the overall metabolic profiles, mitochondrial respiration and glycolysis, were examined by detecting the oxygen consumption rate (OCR) and the extracellular acidification rate (ECAR) *in situ*, respectively. In A549 cells, NSC290956 decreased the initial mitochondrial respiration induced by oncogenic KRas in a dose-dependent manner following the obviously reduced basal respiration and the maximal respiration capacity (Figure 6A). In contrast, glycolysis and glycolysis capacity were almost unchanged with the same concentrations of NSC290956 (Figure 6B). In addition, the metabolic regulation of NSC290956 was applicable to the other cell line with KRasG12C mutant H358 in a similar manner (Figures 6C,D). These results showed that NSC290956 produced a sustained decrease in mitochondrial metabolism. This provides a solid basis for the notion that mitochondrial metabolism is essential for KRas-mediated tumor (Weinberg et al., 2010).

### NSC290956 Altered Mitochondrial Function for Apoptosis

**FIGURE 7:**
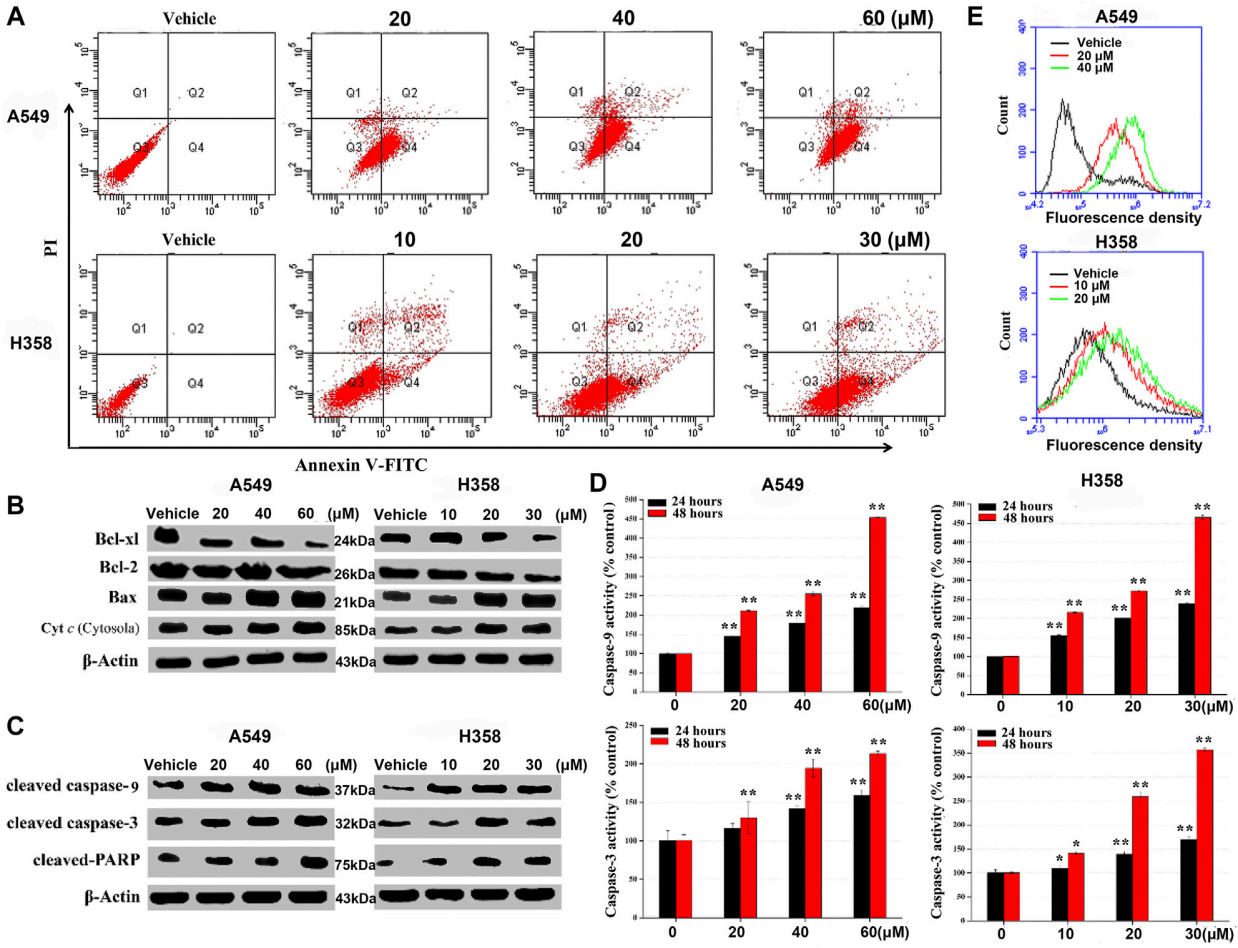
NSC290956 regulates mitochondrial dysfunction for apoptosis in both A549 and H358 cells. **(A)** Apoptosis induced by the indicated dose of NSC290956 was measured by flow cytometry in both the cell lines, respectively. **(B)** Appearance of apoptosis markers in the cells was changed in NSC290956. Effects of NSC290956 on the activation of caspase-3 and caspase-9 were assessed by WB analysis **(C)** and caspase activity assay **(D)**, respectively. β-Actin was used as the internal control. Significant differences from untreated control were indicated *p < 0.05 and **p < 0.01. **(E)** Flow cytometric detection of ROS generation is shown. X axis-fluorescence density; Y axis-cell amount.

To validate if metabolic alterations may be responsible for cell proliferation inhibition, the events related to cell death were monitored as the readout of apoptosis. Flow cytometric data showed a dose-dependent apoptosis-induction effect of NSC290956 on both the cell lines, while the number of necrotic cells among all the cells remained small (Figure 7A; Supplementary Figure S6A). The apoptotic cell death was visually confirmed by cell imaging (Supplementary Figure S6B). WB analyses showed that the anti-apoptotic proteins Bcl-xl and Bcl-2 were remarkably decreased, but the pro-apoptotic protein Bax was significantly increased (Supplementary Figure S7). In addition, NSC290956 obviously promoted the release of cytochrome *c* into cytosol (Figure 7B). The released cytochrome *c* accelerated caspase-9 activation, eventually triggering caspase-3 activation (Figures 7C,D). As an additional response mechanism, the mitochondrial membrane potential (MMP), an important indicator of mitochondrial health, was significantly decreased (Supplementary Figure S8) and the cellular reactive oxygen species (ROS) levels were markedly increased in a dose-dependent manner upon NSC290956 treatment (Figure 7E). Consistent with the decreased mitochondrial metabolism, these results suggested that NSC290956 caused mitochondrial dysfunction. Thus, we propose that NSC290956-mediated cell death likely results from the mitochondria-dependent apoptosis.

### NSC290956 Reduced Tumor Growth in H358 Xenograft

**FIGURE 8:**
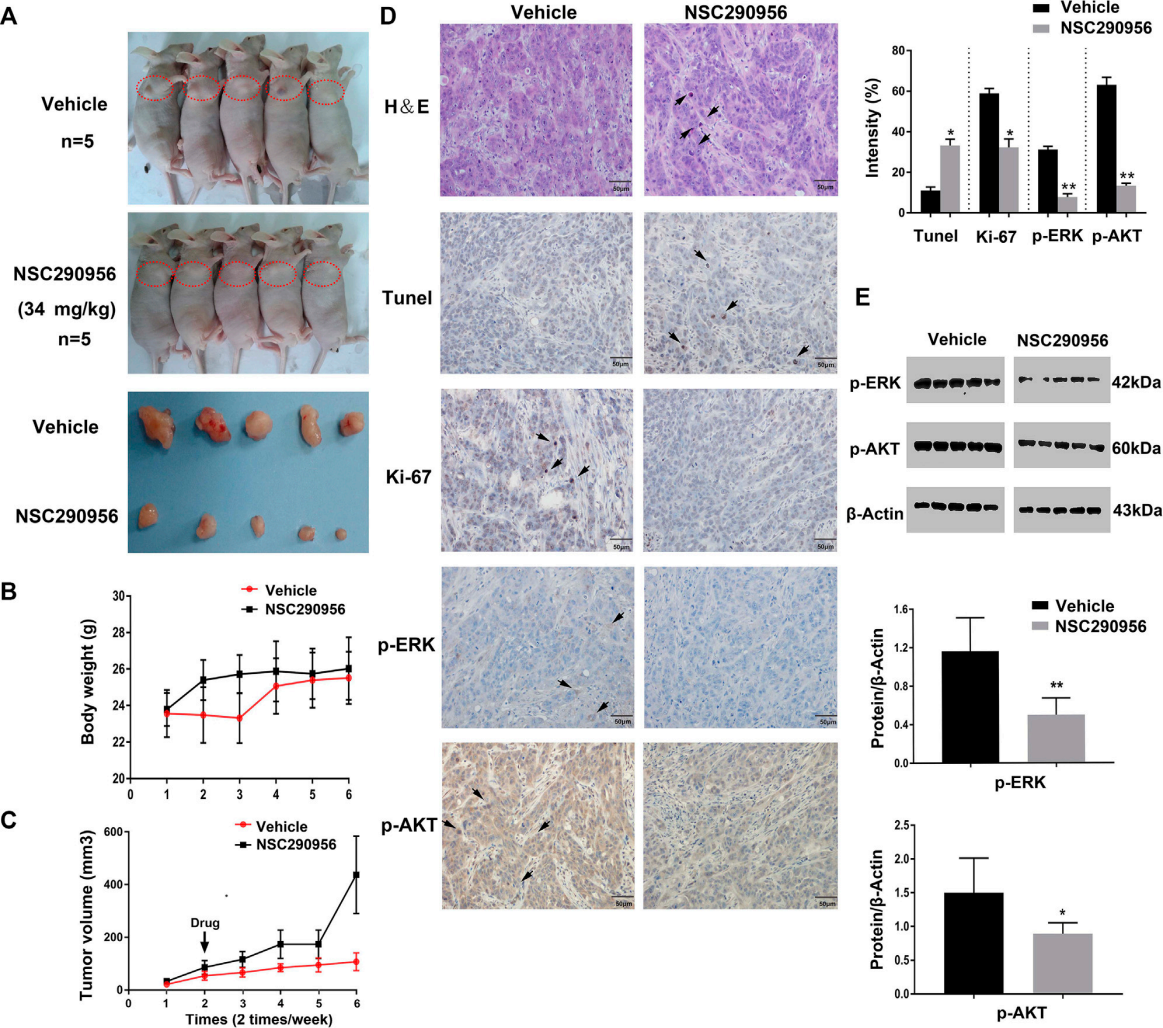
NSC290956 shows activity in the KRas mutant cell line xenograft. **(A)** NSC290956 reduces tumor growth in H358 tumor model. The right anterior shoulder subcutaneous site (red dotted circle) was chosen for xenografting in BALB/c nude mice. The curves of tumor weight **(B)** and tumor volume **(C)** are shown as the mean of five mice in each group ± SEM. The tumor body weight and volume per mouse was respectively recorded every other day. **(D)** Tumor sections were evaluated by using H&E, Tunel, Ki-67, and IHC staining. Shown images are representative sections of the necropsy samples from NSC290956 and vehicle-treated mice. The black arrows show the positive staining. **(E)** Tissue samples taken from mice by western blotting for phosphorylated ERK and AKT.

We next addressed whether the target engagement achieved in the cell can translate to anti-tumor activity in the animal. The *in vivo* efficacy of NSC290956 was initially assessed in xenograft tumor model using H358 (KRasG12C) cells in nude mice. Based on previous renal capsule models of pancreatic cancer MIA PaCa-2 (KRasG12C) cells in BALB/c mice (Guo et al., 2018), NSC290956 was administered by intraperitoneal injection with 34 mg/kg once per every day. The administration effectively reduced tumor growth for the duration of 2 weeks of treatment as compared to vehicle control (Figures 8A–C).

H&E (hematoxylin and eosin) and Ki-67 staining of tumor sections displayed a good cellularity and fewer Ki-67 positive cells compared to vehicle-treated cells (Figure 8D), revealing the efficacy after drug treatment in mice. Likewise, TUNEL staining in tumor sections showed a prominent increase of apoptotic cells in NSC290956-treated mice, which validated a pro-apoptotic contribution of NSC290956 to tumor growth inhibition. To confirm that the tumor growth inhibition is associated with inhibiting KRas signaling *in vivo*, these tumor samples were analyzed for markers of pharmacological KRas inhibition. Compared to vehicle-treated mice, the decreased phosphorylation levels of ERK and AKT were observed after NSC290956 administration. These results suggest that the *in vivo* efficacy of NSC290956 is related to the attenuations of MAPK-ERK and AKT-mTOR dual signaling (Figure 8E). Together, these results effectively demonstrated the treatment effectiveness of NSC290956 in the mutant KRas-driven NSCLC.

## Discussion

More than one-third of human tumors harbor active Ras mutations (Prior et al., 2012). Abnormal activation of Ras signaling in tumor indicates that it might be a key tumor target. These findings motivated extensive efforts to target the oncoproteins for tumor therapy. Regrettably, attempts to directly inhibit the oncogenic Ras came with little success (Ostrem and Shokat, 2016).

Extensive efforts have focused on targeting individual sites on the surface of Ras proteins by structure-based computational screening. Stockwell et al. applied a computational design approach to yield a multivalent pan-Ras inhibitor (compound 3144) that exhibited cellular lethality, partially dependent on Ras expression (Welsch et al., 2017). Liu et al. described a computational structure-based design and identification of ARS-1620, a covalent compound with high potency and selectivity for KRasG12C mutant cancers (Janes et al., 2018). Kataoka group reported the successful development of Kobe0065-family compounds by an *in silico* screening targeting a pock on M-Ras-GTP (Shima et al., 2013). These compounds may serve as scaffolds for the development of Ras inhibitors with higher potency and specificity. Here, we took advantage of the SPA screening approach to characterize and tackle the druggability of the “open conformation” with both affinity and specificity, which relies on the combination of *in silico* calculations, pharmacophore modeling, and molecular docking. The immediate outcome was the discovery of hit candidate NSC290956 for the first time. Besides NSC290956, other two compounds NSC48693 and NSC48160 had been identified to promote apoptosis in human pancreatic cancer cells harboring oncogenic KRas by the Figure 1 workflow. NSC48160 inhibited cell viability and colony formation in CFPAC-1 (KRasG12V) and BxPC-3 (wild-type KRas) cell lines (Li et al., 2013). NSC48693 had an ability to block the anchorage-independent growth of CFPAC-1, MIA PaCa-2, and BxPC-3 cancer cells in soft agar (Liu et al., 2012).

Given the predominant role of oncogenic KRas in tumor, therapeutic strategy either targeting the mutated KRas or inhibiting the excessive KRas signaling is valuable in the treatment of tumors. Targeting the “open conformation” therefore represents a breakthrough progress for developing a new KRas-targeted anti-tumor strategy. In theory, NSC290956 serves to bind to KRas and then to diminish the activated KRas-GTP level. The current results revealed the KRas engagement of NSC290956 in intact cells by thermally stabilizing KRas (Figure 3D), further blocking the cellular KRas activity (Figure 4) and resulting in a degree of KRas-dependent lethality in NSCLC tumor cells (Figure 5A). Especially, it is worth noting that there are multiple relevant targets in cancer cells. It is impossible to exclude the possibility that NSC290956 blocks the KRas signaling events due to the unspecific interactions with other proteins. This will be explored in further work. NSC290956 at least manifested potent efficacy against the inhibition of KRas-mediated signaling, resulting in the dramatically reduced survival of the KRas-expressing human pancreatic cancer cell lines, including CFPAC-1 (KRasG12V), MIA PaCa-2 (KRasG12C), Capan-1 (KRasG12V), SW1990 (KRasG12T), and BxPC-3 (wild-type KRas) with IC_50_ ranging 19.7–74.2 μM. However, little significant cytotoxicity was observed for human normal cell lines HEK293, HL7702, and PBMC with IC_50_ ranging 71.2–125.6 μM. In this respect, targeting the “open conformation” may offer a much higher therapeutic index for selectively killing tumor cells while sparing healthy ones because of the discriminated inhibition of both cancerous and non-cancerous cells.

The interplay between metabolic remodeling and oncogenes has being appreciated in tumor. It is still elusive to understand the underlying mechanism for metabolic switching between cancer glycolysis and mitochondrial respiration, though progress has been made in this issue. Recently, Li and Wang developed an integrative network model to uncover the core role of oncogene Ras in the gene regulatory network and cancer-related metabolic pathway (Li and Wang, 2020). *In silico* calculations predicted that oncogenic KRas inactivation is a key factor for metabolic switching between mitochondrial and glycolytic metabolism in cancer. Herein we provide evidences that inactivating KRas with NSC290956 modifies metabolic features of KRas mutant A549 and H358 cells imposing repression of mitochondrial respiration without significant compensatory increase in glycolysis (Figure 6). The distinctive characteristics of the specific cell metabolism are in support of the view that disrupting the tumor metabolic phenotype can be exploited to preferentially kill the cancer cells. Obviously, the cytotoxic selectivity of NSC290956 between cancerous cells and normal cells as shown in Figure 5B can attribute to the characteristics of specific cell metabolism. This finding extends the current perspective that mitochondrial metabolism is essential for KRas-mediated tumor (Weinberg et al., 2010). These data indicate that the dysregulation of particular mitochondrial metabolic pathway may play a pivotal role in dictating which adaptive mechanism is therapeutic index in the treatment of a KRas-positive tumor. Further study is ongoing to enlarge this evidence on other KRas-mutated cancer cells with the final aim to evaluate whether NSC290956 can be a suitable therapeutic agent targeting metabolic vulnerability.

Following the *in vitro* observations in KRas-mutant NSCLC cells, the effect of NSC290956 was further evaluated in a subcutaneous tumor model using H358 tumor xenograft (Figure 8). In the case of BALB/c nude mouse model, NSC290956 treatment led to the dramatically decreased tumor growth. The initial toxicity study *in vivo* showed no major toxicity using a chronic injection of NSC290956 into BALB/c nude mice, as it displayed that NSC290956 did not cause significant alteration in body weight loss. Guo et al. reported that Spiclomazine (i.e., NSC290956) more effectively reduced the tumor growth of MIA PaCa-2 in renal capsule xenografts than Gemcitabine-based treatment (Guo et al., 2018). Notably, in three of the five mice, the growth of tumors after Spiclomazine treatment was completely blocked. Especially, all of the Spiclomazine-treated mice did not show any body weight loss and other obvious signs of toxicity, and they were alive and healthy throughout the study during the treatment. In contrast, the Gemcitabine-treated mice showed a marked body weight loss and even death. These findings were in good agreement with the previous study that selective blocking of the activated KRas inhibited KRas transformation in animal models (Podsypanina et al., 2008). In NSC290956-treated mice, the ERK and AKT phosphoproteins in tumor sections were markedly decreased, which confirmed that the *in vivo* efficacy of NSC290956 is correlated with the impairment of KRas-mediated signaling. Together, the identification of NSC290956 determining the *in vivo* response to KRas is therefore timely for developing a better strategy to treat KRas-positive tumors.

## Conclusion

There have been many attempts to simultaneously inhibit MAPK-ERK and AKT-mTOR dual pathways using a combination of inhibitors, which have generally failed to show an acceptable therapeutic index due to combined toxicity (Ostrem and Shokat, 2016). Our studies indicate that NSC290956 as a single agent shows a remarkable safety profile in spite of inhibiting dual signaling, which contributes to its unique mechanism of action. The current results demonstrate that drug discovery based on blocking Ras “open conformation” may be complementary to the therapeutic strategy of direct targeting Ras. This work bridges the gap between *in silico* virtual drug screening and proof-of-concept pre-clinical testing.

## Methods

### Materials and Reagents

NSC290956 was dissolved in DMSO to make stock solution (10 mg/ml) and further diluted to appropriate concentrations with double distilled water containing 10% DMSO immediately before use. Dulbecco’s phosphate buffered saline (PBS) was purchased from Sigma-Aldrich Inc. (St. Louis, MO). AnnexinV-propidium iodide (PI) apoptosis detection reagent was purchased from BD Bioscience (San Jose, CA). In addition, 5-(and 6)-chloromethyl-2′,7′-dichlorodihy-drofluorescein diacetate (CM-H2DCFDA) and 4′,6-Diamidino-2-phenylindole (DAPI) were purchased from Invitrogen (Carlsbad, CA). Western blotting substrate was obtained from Thermo Scientific (Hudson, NH). Unless otherwise notified, all chemicals were obtained from Sigma-Aldrich. Polyvinyl-idenedifluoride (PVDF) membrane was purchased from EMD Millipore (Bedford, MA). Media (RPMI-1640 and DMEM) and fetal bovine serum (FBS) were purchased from Gibico (Grand Island, NY). Primary antibodies against human CRAF, MEK, phospho-MEK, ERK1/2, phospho-ERK1/2, AKT, phospho-AKT, phospho-PI-3K, phospho-mTOR, mTOR, Bcl-xl, Bcl-2, Bax, cytochrome c, cleaved caspase-9 and caspase-3, cleaved PARP, CDK1, Cyclin B1, Beclin1, KRas (C-terminal), and β-actin were all from Abcam Technology Inc. (Beverly, MA). The horseradish peroxidase-conjugated anti-rabbit or anti-mouse secondary antibodies were purchased from Santa Cruz Biotechnology Inc. (Santa Cruz, CA). Seahorse kits were purchased from Agilent.

### Cell Culture

Human non-small cell lung carcinoma (H358 and A549) and pancreatic cancer (CFPAC-1, MIAPaCa-2, BxPC-3, SW1990, and Capan-1) cell lines were obtained from American Type Culture Collection (ATCC, Rockville, MD) and cultured in RPMI-1640 and DMEM or IMDM media supplemented with 10% FBS and 100 units/ml penicillin, 50 µg/ml streptomycin, and 100 µg/ml amphotericin (Invitrogen, Carlsbad, CA) at 37°C in 5% CO_2_ incubator, respectively. Human normal embryonic kidney (HEK-293), liver (HL-7702), and peripheral blood mononuclear cell (PBMC) cells were purchased from Chinese Academy of Science Type Culture Collection (Shanghai, China) and incubated in DMEM media containing 10% FBS. All the cells were maintained in 25 ml flasks and kept in an atmosphere of 5% CO_2_ and 95% air under humidified conditions at 37°C. The adherent cells other than PBMC were detached from the monolayer using 0.25% trypsin and 0.53 mM EDTA for 5 min at 37°Cwhen cells were grown to near confluence. All the cell lines were tested for *mycoplasma* contamination by using *mycoplasma* stain assay kit (Beyotime Institute of Biotechnology, China).

### SPecificity-Affinity Virtual Screening

SPA, a two-dimension virtual screening strategy, is promising in searching for po-tential compounds due to its simultaneous reaching performance maximization on af-finity and specificity (Wang et al., 2007). HRasG60A-GppNp (PDB: 1XCM [Matsunaga et al., 2017], GppNp, Guanosine-5’ -[(β,γ)-imido] triphosphate, a non-hydrolysable form of GTP) was used as the docking receptor in in silico calculations using Autodock3 package of programs (Morris et al., 1998). The cavity located between switch 1 region and residues Ile21, Gln25, Asp30, Tyr32, and Pro34 is defined as the binding pocket. The solvent parameters and partial charges were generated from the PRODRG 2 server (Schüttelkopf and van Aalten, 2004). GppNp and cofactor Mg^2+^ were retained for screening ligand. The water molecules interacting with Mg^2+^ were also retained. The other water, organic solvent, and lipid molecules were removed. Protons were automatically assigned in such a way that the protonation states of side chains and termini corresponded to pH 7.0. The *de novo* designs of compounds in the National Cancer Institute (NCI) database were carried out using the autogrow package according to scaffold of potential inhibitors (Zheng et al., 2013a; Zheng et al., 2013b). The ADME/TOX filtering was carried out via the online FAF-Drugs2 tool (Lagorce et al., 2011). Pharmacophore models were constructed for compound using the LigandScout with default parameters (Wolber and Langer, 2005). The clustering analysis was carried out by AuPosSOM program (Bouvier et al., 2010).

In brief, SPA screening process for the “open conformation” was performed as displayed in the work flow (Figure 1). The affinity was calculated by measuring free energy difference before and after association. The specificity was calculated by measuring the gap δE (difference between the lowest energy and the average energy) and the spread or the variance of the energy spectrum ΔE (Wang et al., 2007). We defined the intrinsic specificity ratio (ISR) = δE/ΔE. Maximizing ISR is an optimization criterion for the binding specificity (Yan et al., 2013). Large ISR implies that the lowest energy (native binding) state is stable, significantly discriminated from the rest of the binding modes, and therefore specific. Since affinity may not always correlate with specificity, which is the ultimate goal for inhibitor design, ISR is used as an additional dimension for screening. Thus, compounds that had consensus free energy (high affinity) between −10 and −8 kcal/mol as well as ISR value between 4 and 7.5 were chosen from the database. Finally, 26 hit candidates with high affinity and high specificity were selected and presented from NCI for next bioassay test. The most promising hit NSC290956 (also termed APY606 or Spiclomazine) was chosen for the final evaluation due to its minimum IC_50_ value.

### Confirmation of Protein by HPLC

A 3.0 μl aliquot of protein supernatant was injected using the Waters 1525 Binary HPLC Pump equipped with Waters 2998 Photodiode Array Detector. Mobile phase: 2 mM ammonium formate aqueous solution containing 0.05% formic acid (buffer A) and methanol (buffer B). Flow rate: 500 μl/min. Elution protocol: linear gradients (0–1.0 min from 80% A/20% B to 50% A/50% B, 1–1.5 min 50% A/50% B, 1.5–1.6 min from 50% A/50% B to 80% A/20% B, 1.6–3 min is 80% A/20% B).

### Western Blotting Analysis

To clarify the underlying mechanisms of NSC290956 bioactivity at the molecular level, WB analysis was examined. H358 and A549 cell lines were seeded onto 100 mm plates. After being treated by NSC290956 as indicated concentrations, cells harvested were washed with cold PBS. After cellular proteins were extracted by using protein lysis kit (KeyGEN Biotech, Nanjing, China), protein concentrations were measured by Pierce BCA protein assay kit. Equal amount of total cellular protein (30 μg) was separated on 10–12% SDS-PAGE gel and then transferred onto PVDF membrane. Thereafter, membrane was blocked in 5% nonfat dry milk in TBST and probed with the relevant primary antibody (1:1,000 dilution) overnight at 4°C. Visualization was performed using the appropriate horseradish peroxidase-conjugated secondary antibody (1:1,000 dilution). The specific proteins were scanned by using Chemiluminescence detector (DNR, KiryatAnavim, Israel). Protein level was normalized to the matching densitometric value of the internal control β-Actin.

### Cellular Thermal Shift Assay

The ability of NSC290956 to interact with and stabilize KRas proteins in intact cells was analyzed essentially by CETSA. Briefly, H358 and A549 cells were grown in 100 mm plates to 70–80% confluency and then treated with cell media containing 0.1% DMSO and 12.5 µg/ml NSC290956 for 8 h, respectively. After treatment, cells harvested were collected by centrifugation and subsequently resuspended in TBS buffer. Thereafter, these cells were suspended in lysis buffer and incubated on ice for 20 min. Cells were then centrifuged at 14,000 rpm for 15 min at 4°C. The cell suspension was placed into microtubes and heated for 3 min to 44, 46, 48, 50, 52, 54, 56, 58, and 60°C followed by cooling for 3 min at room temperature. Precipitates were separated from the heated lysates by centrifugation at 14,000 rpm for 20 min at 4°C. The soluble protein super-natants were transferred to new microtubes and analyzed by SDS-PAGE followed by WB analysis using KRas (C-terminal) antibody at a concentration of 1:500.

### Cell Viability Assay

The cell viability was assessed by Cell Counting Kit-8 (CCK-8) assay kit (Dojindo, Japan). Briefly, the cells seeded onto 96-well plates were treated by double distilled water containing DMSO as vehicle control groups or various concentrations of NSC290956 as experimental groups. Each concentration of a group of drugs was set five alternative holes. The cells were treated for 24 or 48 h and then the optical density (OD) at 450 nm was read with a M200 PRO NanoQuantautoreader (TECAN, Switzerland). The final concentration of DMSO in the culture media is 0.1% (v/v), which does not significantly affect the cells.

### Flow Cytometric Detection of Apoptosis

Based on the growth cycle of cancer cells, the cells were treated by NSC290956 for 24 h in order to discriminate the cell death as caused by either apoptosis induction or necrosis. Apoptotic H358 and A549 cells were quantified by Annexin V-fluorescein isothiocyanate (FITC)-propidium iodide (PI) apoptosis detection kit after cells were treated with double distilled water containing DMSO (final concentration of 0.1%) as vehicle control groups or various concentrations of NSC290956 as experimental groups for 24 h. Briefly, cells were trypsinized and washed twice with cold PBS, and then the cells were resuspended at a density of 1 × 10^6^ cells/mL in binding buffer. Thereafter, a quota of the cells were gently mixed and incubated in a 1.5 ml EP tube with 5 µl annexin V-FITC and 5 µl PI in the dark for 15 min at room temperature. A quota of binding buffer was then added to each tube and the cells were subjected to flow cytometric analysis (Accuri C6, Ann Arbor, MI). In total, 10,000 events were analyzed in each sample. Cells that stain positive for FITC annexin V and negative for PI are undergoing apoptosis (Q4 quadrant); cells that stain positive for both FITC annexin V and PI are either in the end stage of apoptosis (undergoing necrosis) (Q1 quadrant) or already dead (Q2 quadrant); and cells that stain negative for both FITC annexin V and PI are alive and not undergoing measurable apoptosis (Q3 quadrant).

### Apoptotic Nuclear Staining

NSC290956-induced apoptotic nuclear condensation and morphological change were detected using DAPI staining. H358 and A549 cell lines were grown on glass-bottom plates to 50% confluence and then cultured in the medium in the presence of various concentrations of NSC290956 for 24 h. Thereafter, the cells were fixed with 3.5% paraformaldehyde and then incubated in a fluid containing 2 mg/ml DAPI for 20 min. The nuclear morphology of cells was observed by fluorescence microscopy (AMG EVOS, Life).

### Fluorescence Imaging of Cell Apoptosis

Apoptosis of H358 and A549 cells seeded onto glass-bottomed plates was qualitatively measured using fluorescence microscopy (AMG EVOS, Life) at 488 and 543 nm to assess the FITC and PI signals. Cells that stain positive for FITC annexin V and negative for PI are undergoing apoptosis; cells that stain positive for both FITC annexin V and PI are either in the end stage of apoptosis (undergoing necrosis) or already dead; and cells that stain negative for both FITC annexin V and PI are alive and not undergoing measurable apoptosis.

### Measurement of Mitochondrial Membrane Potential (ΔΨm)

Disruption of ΔΨm is a characteristic signature of apoptosis in mitochondrial-related pathway. ΔΨm can be measured using fluorescent probe Rhodamine 123. Briefly, H358 and A549 cell lines were seeded into 6-well plates and treated with NSC290956 at different concentrations for 24 h. Cells were washed with PBS and incubated in 1 mM Rhodamine 123 solution for 20 min. Thereafter, cells were resuspended in 500 µl PBS buffer and subject to flow cytometer (Accuri C6, Ann Arbor, MI). In total, 10,000 events were analyzed in each sample. The decrease of fluorescence intensity indicates the loss of ΔΨm in cancer cells.

### Measurement of Intracellular Reactive Oxygen Species Level

Intracellular ROS level was measured by flow cytometric analysis as described previously. In brief, cells (5 × 10^4^ cells/well) were grown in 24-well plates at 5% CO_2_ and 37°C conditions. After treatment with vehicle or various concentrations NSC290956 for 24 h, the cells were incubated with 5 μM CM-H2DCFDA in PBS for 30 min and subjected to flow cytometry (Accuri C6, Ann Arbor, MI) to determine ROS levels at wavelength of 488 (excitation) and 530 (emission). In total, 10,000 events were analyzed in each sample.

### Cell Cycle Analysis

To test the potent mechanism for NSC290956-induced cell growth inhibition, the effect of NSC290956 treatment on cell cycle distribution was explored by flow cytometry. Based on the duration of the cell cycle, it is feasible and reasonable to detect the cell cycle progression at the cell cycle checkpoint when cells are treated for 24 h. In brief, H358 and A549 cell lines were seeded into six-well plates and treated with NSC290956 at the indicated concentrations for 24 h, respectively. Cells were suspended and fixed in 70% (v/v) ethanol at 4°C overnight. Thereafter, cells were washed with PBS, resuspended in 1 ml of PBS containing 50 µg/ml PI and 1 mg/ml RNaseA at room temperature in dark for 30 min. In total, 10,000 events were analyzed immediately in each sample by flow cytometer (Accuri C6, Ann Arbor, MI). All experiments were repeated at least three times.

### Caspase Activity Assay

Both H358 and A549 cells seeded in 6-well plates (5% CO_2_, 37°C) were pre-incubated overnight before NSC290956 was added to each well. After a period of exposure (24 or 48 h) with various concentrations of NSC290956, cells harvested by trypsinization were washed by PBS and the activity of caspase-3 and caspase-9 was determined by caspase activity assay kit from Abcam Technology Inc. (Beverly, MA), respectively. The absorbance of enzymatically released pNA was measured at 490 nm on a M200 PRO NanoQuantautoreader (TECAN, Switzerland).

### Tumor Xenograft Models

Femal athymic nude mice (6–8 weeks of age) were allowed to acclimate for 2 weeks in sterile micro isolator cages with constant temperature and humidity, with simultaneous free access to food and water 2 × 10 (Ford et al., 2005). H358 cells suspended in the fresh medium containing 10% Matrigel were injected subcutaneously per mouse. Tumors were allowed to grow to ∼50 mm^3^, and then mice were randomized into two groups (five per group). Mice were monitored daily and weighed twice weekly. Drug treatment started the day after inoculation. NSC290956 dissolved in DMSO was injected intraperitoneally every 2 days at 34 mg/kg for 2 weeks. DMSO with the same final concentration was injected intraperitoneally as vehicle-treated negative control. Mice were sacrificed at the end of the second week and the tumor from each mouse was excised. A portion of the tumors was fixed and processed for paraffin embedding and immunohistochemical (IHC) analysis. This animal protocol used in the current study was in accordance with the recommendation of the Chinese Academy of Sciences Ethics Committee and under the supervision of authorized investigators.

### Statistical Analysis

All datas were expressed as mean ± SD of triplicate experiments, and reproducibility was confirmed in at least three separate experiments. Statistical analysis was performed by using GraphPad Prism Software version 7.04 (GraphPad, CA, United States). *p < 0.05 and **p < 0.01 were considered statistically significant.

## Data Availability

The original contributions presented in the study are included in the article/Supplementary Material, and further inquiries can be directed to the corresponding authors.
