# Supplementary material for: Discovery of Small Molecule NSC290956 as a Therapeutic Agent for KRas Mutant Non-Small-Cell Lung Cancer
Source: Front Pharmacol. 2022 Jan 5;12:797821. doi: 10.3389/fphar.2021.797821 (PMC8766838; doi:10.3389/fphar.2021.797821)
Supplement: Supplementary file 1 [file Table1.DOCX]

Discovery of small molecule NSC290956 targeting the open conformation of Ras as a therapeutic agent for non-small-cell lung cancer

Jiaxin Zhang^1, 2^, Zuojia Liu^1,*^, Wenjing Zhao^1^, Xunzhe Yin^1^, Xiliang Zheng^1^, Chuanbo Liu^1^, Jin Wang^3,*^ and Erkang Wang^1,2,*^

^1^State Key Laboratory of Electroanalytical Chemistry, Changchun Institute of Applied Chemistry, Chinese Academy of Sciences, Changchun, Jilin, China.

^2^University of Science and Technology of China, Hefei, Anhui 230026, China.

^3^Department of Chemistry and Physics, State University of New York 11794-3400, Stony Brook, New York, USA.

^*^Correspondence:

[zjliu@ciac.ac.cn; Tel.:+86-431-85262914](mailto:zjliu@ciac.ac.cn;%20Tel.:+86-431-85262914);

[jin.wang.1@stonybrook.edu](mailto:jin.wang.1@stonybrook.edu); Tel.:+1-631-632-1185;

[ekwang@ciac.ac.cn](mailto:ekwang@ciac.ac.cn); Tel.: 86-431-85262003.

**Synthesis of NSC290956**

NSC290956 was synthesized by the following route:

*Synthesis of compound* ***1***

A mixture of 1-benzylpiperidin-4-one (1.95 g, 10.3 mmol), thioglycolicacid (8.08 g, 87.7 mmol), ammonium carbonate (8.83 g, 91.8 mmol), and toluene (60 mL) was heated under a water-separator for 20 hr. Then most of toluene was evaporated under reduced pressure. The residue was dissolved in methylene chloride, washed with saturated sodium carbonate (3 x 50 mL), and dried over Na_2_SO_4_. Removal of dichloromethane under reduced pressure and purification by silica gel column chromatography on silica gel afforded **1** as a solid (Yield: 3.241 g, 80%). ^1^H NMR (400 MHz, CDCl_3_) δ 7.66 (s, 1H), 7.40-7.21 (m, 5H), 3.56 (s, 2H), 3.52 (s, 2H), 2.73 (s, 2H), 2.32 (s, 2H), 2.08-1.95 (m, 4H).

*Synthesis of compound* ***2***

To solution **1** (105 mg, 0.4 mmol) in anhydrous THF (10 mL) under nitrogen at -78^o^C was slowly added 1-chloroethyl chloroformate (86 µl, 0.8 mmol). The reactionmixture was stirred for 1 hr then allowed to warm to room temperature. THF was removed under reduced pressure, leaving a residual which was diluted with methanol (10 mL) and heated at reflux for 1 hr. The solvent was evaporated and then the residue was purified by silica gel column chromatography affording **2** as a yellow solid (Yield: 0.727 g, 46%). ^1^H NMR (400 MHz, DMSO-*d_6_*) δ 8.78 (s, 1H), 3.45 (s, 2H), 2.89 (d, *J* = 12.8 Hz, 2H), 2.55 (dt, *J* = 14.6 Hz, 3.6 HZ, 2H) , 1.88 (s, 1H), 1.81 – 1.68 (m, 4H).

*Synthesis of compound* ***3***

Compound 2-chloro-10*H*-phenothiazine (1.196 g, 5 mmol) was dissolved in 3 mL of dry THF with the aid of heat. This solution was added to a suspension of NaH (0.2 g, 5 mmol) in 10 mL of dry DMSO and 5 mL of dry THF. The mixture was stirred under nitrogen at 0^o^C for 30 min, and then was added to a solution of 1-chloro-3-iodopropane (1.08 g, 5.26 mmol) in 2 mL of DMSO. The resulting mixture was stirred at room temperature under nitrogen for 4 h, and was poured into 15 mL of ice water. Extraction with dichloromethane (3 x 15 mL) was dried over MgSO_4_, concentrated under reduced pressure, and purified by silica gel column chromatography provided **3** as an oil (Yield: 0.754 g, 49%). ^1^H NMR (400 MHz, CDCl_3_) δ 7.20 - 7.12 (m, 2H), 7.04 (d, *J* = 8.1 Hz, 1H), 6.97 - 6.85 (m, 4H), 4.04 (t, *J* = 6.4 Hz, 2H), 3.65 (t, *J* = 6.0 Hz, 2H), 2.22 (hept, *J* = 6.4 Hz, 2H).

*Synthesis of* ***4*** *(NSC290956)*

A mixture of **2** (0.34 g, 2 mmol), **3** (0.62 g, 2 mmol), K_2_CO_3_ (0.415 g, 3 mmol), and KI (0.33 g, 2 mmol) in 25 mL of methylethylketone was heated under reflux for 8 hr. After being cooled, the insoluble matter was removed by filtration. The filtrate was concentrated and purified by silica gel column chromatography affording a yellow solid of **4** (Yield: 0.32 g, 67%). ^1^H NMR (400 MHz, CDCl_3_) δ 7.44 (s, 1H), 7.12-7.10 (m, 2H), 7.02-7.00 (m,1H), 6.94-6.84 (m, 4H), 3.89 (t, *J* = 13.2 Hz, 2H), 3.53 (s, 2H), 2.81- 2.57 (m, 2H), 2.47 (t, *J* = 6.4 Hz, 2H), 2.38 - 2.20 (m, 2H), 2.05 - 1.81 (m, 6H).

**ESI-MS confirmation of NSC290956**

The Applied Biosystems 6500 QTrap mass spectrometer equipped with ion spray source was used for NSC290956 confirmation. Peak areas were detected using positive ion mode with the m/z 446.1.


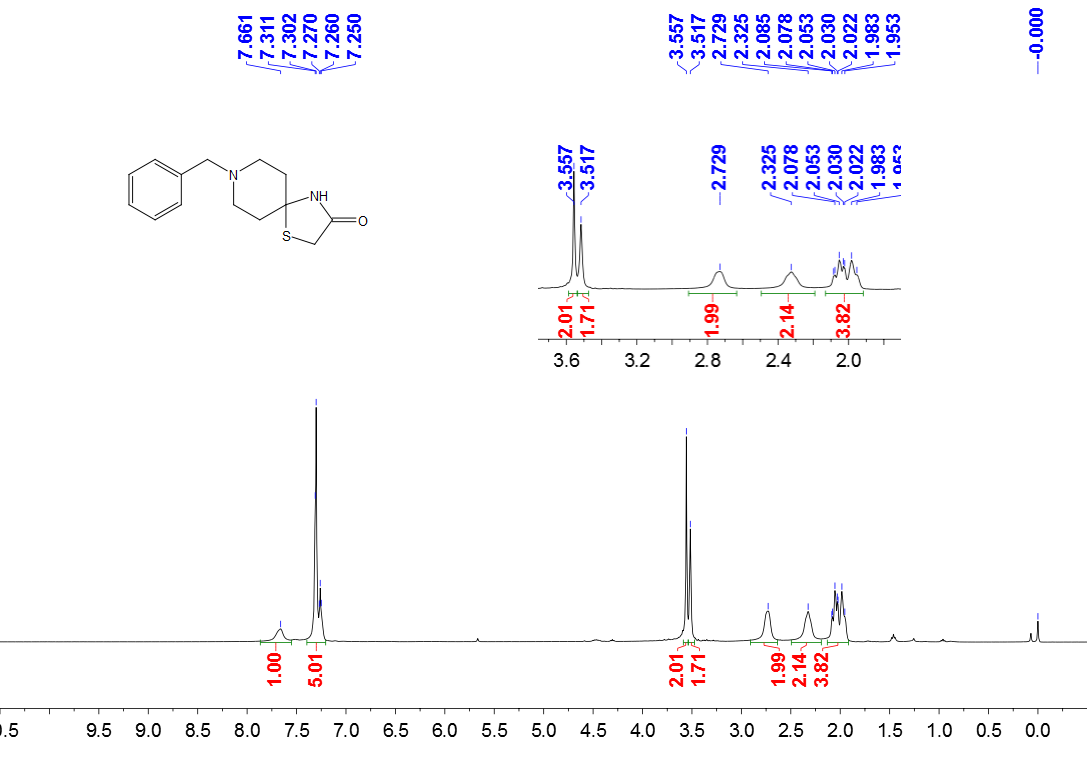


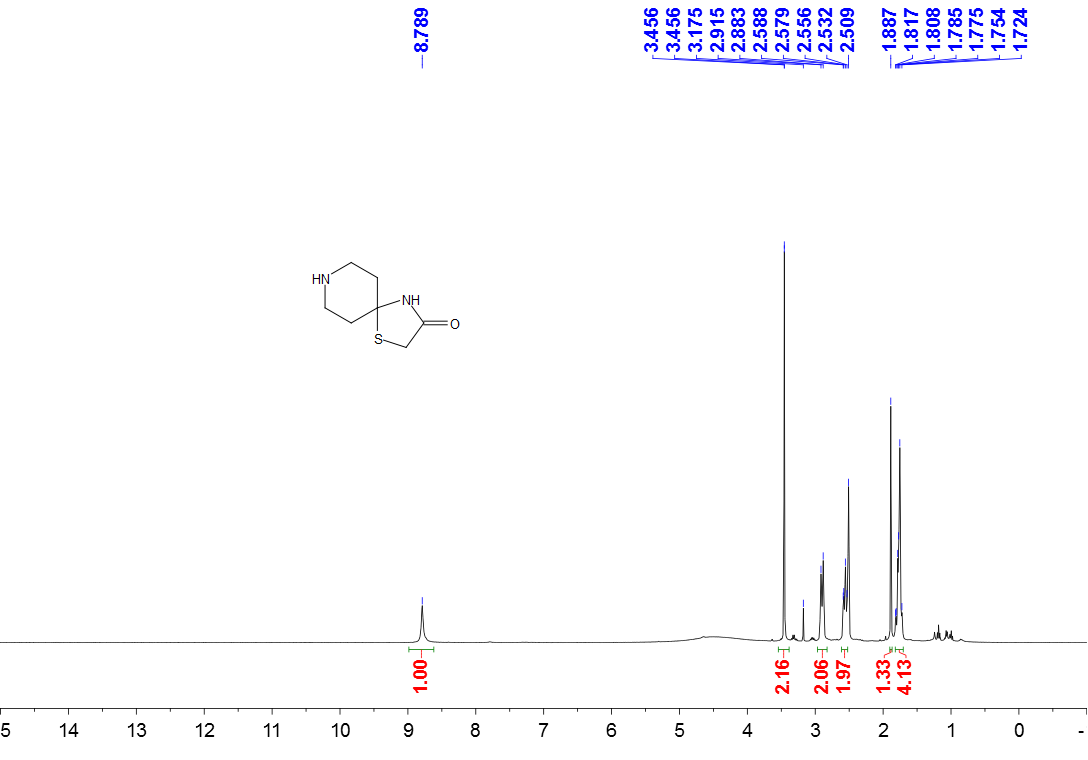


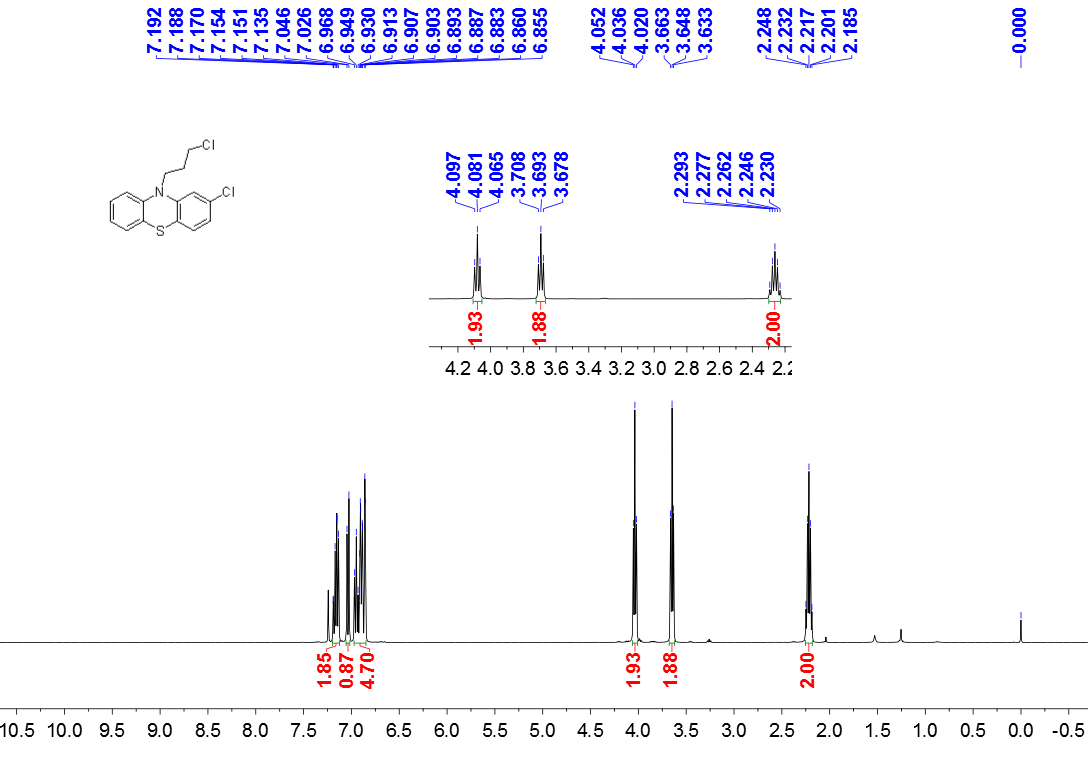


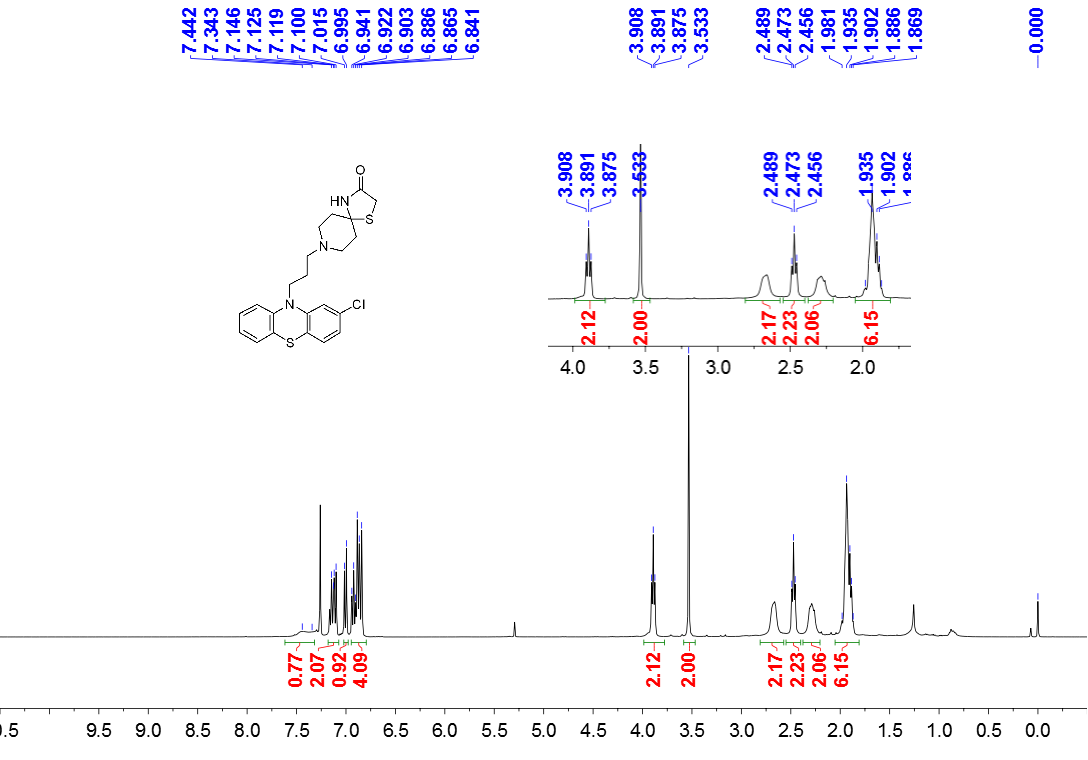


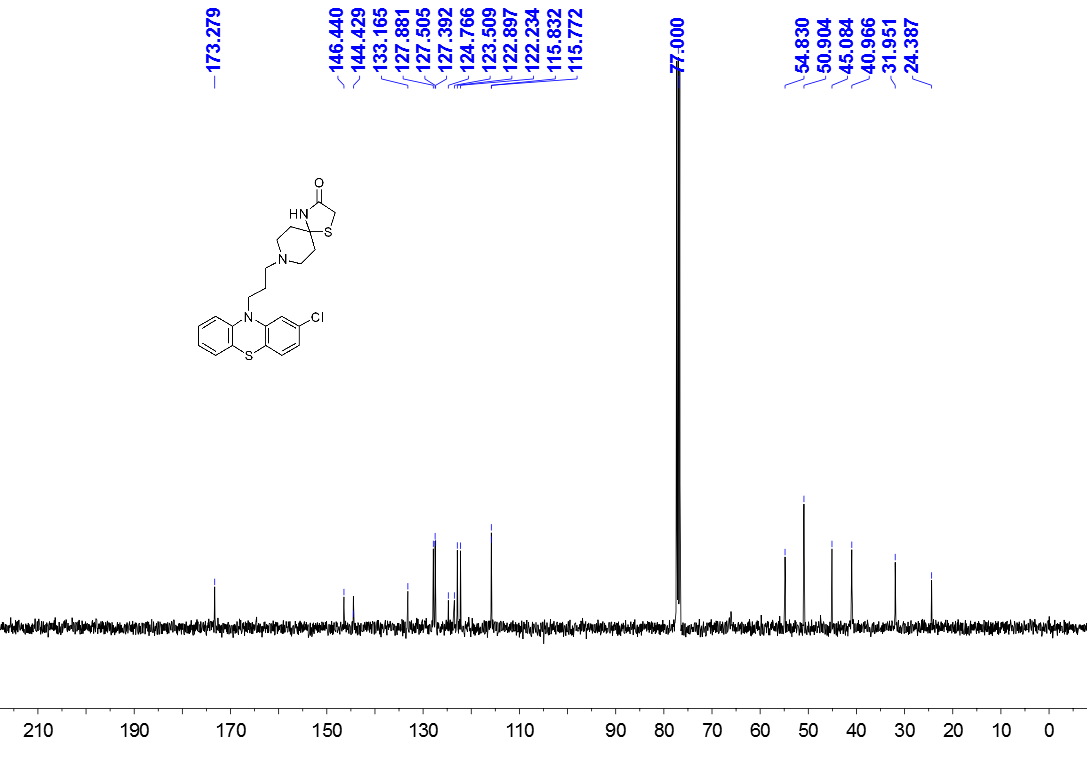


**Supplementary Figure 1****. Related to Figure 2B**. (A) Chemical structure of NSC290956. (B) Confirmation of NSC290956 by using ESI-MS (Molecular Weight: 446.1).

**
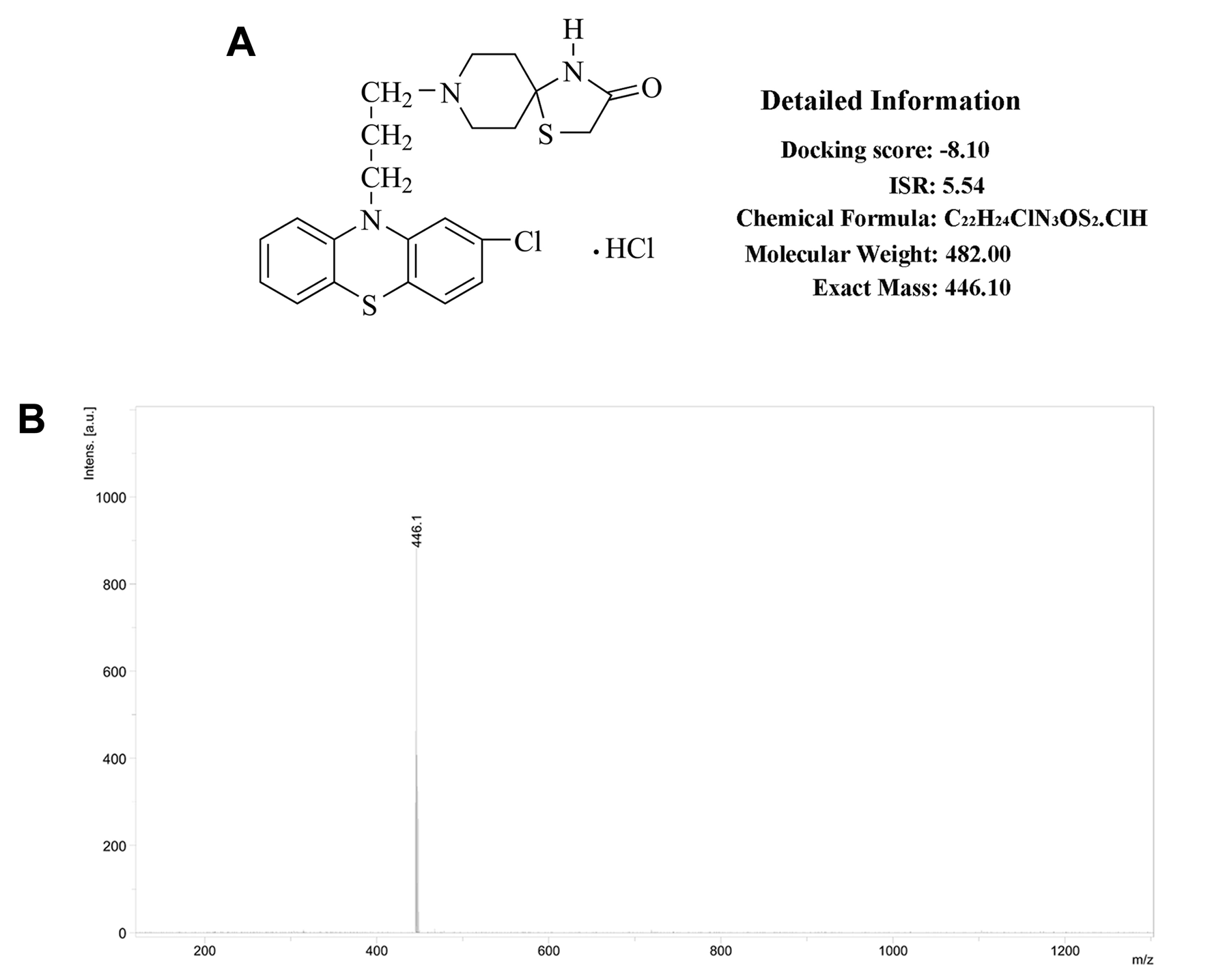
**

**Supplementary Figure 2. Related to Figure 3B**. The response signal (RIU) as a function of time indicates that NSC290956 is unable to bind to HRas-GppNp in SPR experiment.


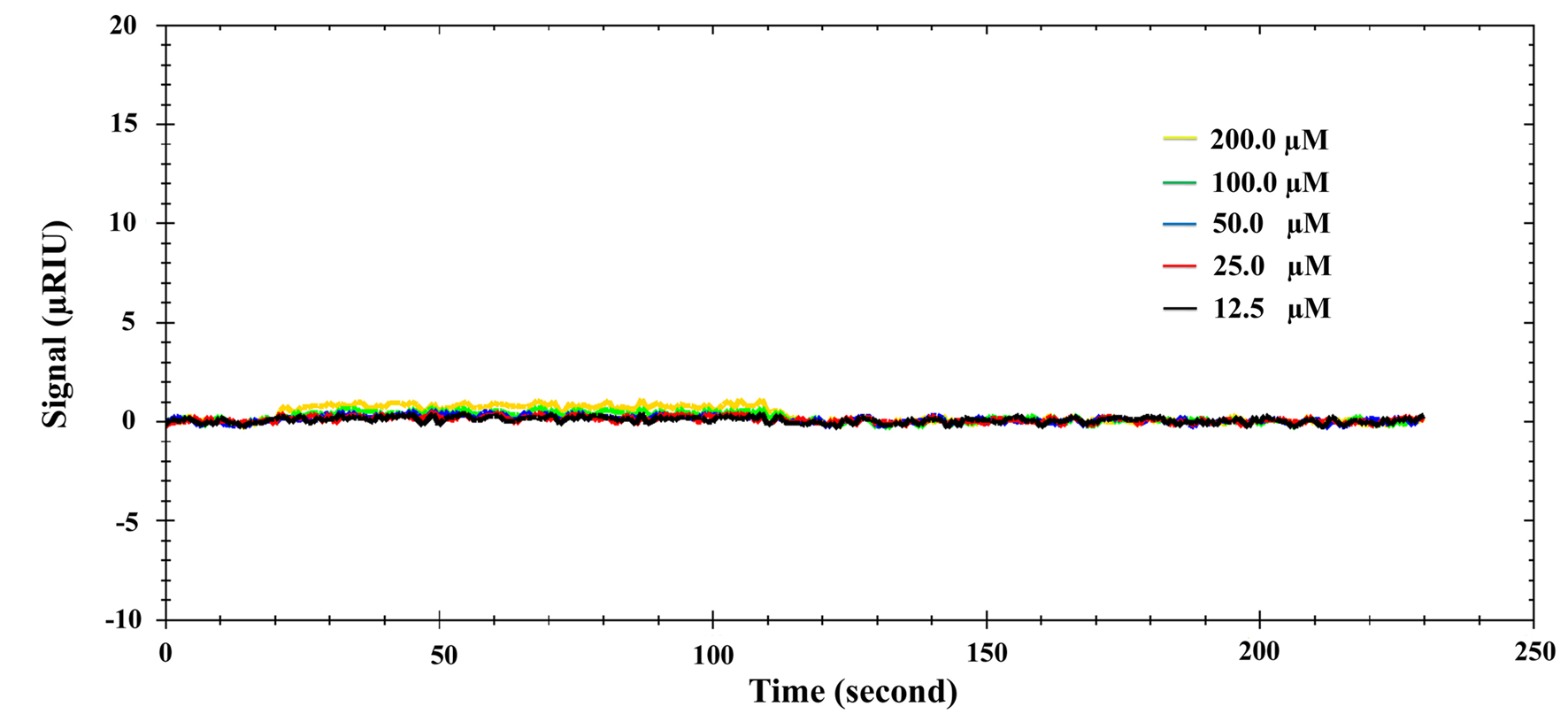


**Supplementary Figure 3. Related to Figure 4.** NSC290956 inhibited KRas activity and downstream signaling events in KRas-driven NSCLC cells. Significant differences from untreated control were indicated ^*^*p*< 0.05, ^**^*p*< 0.01, and ^***^*p*< 0.001.


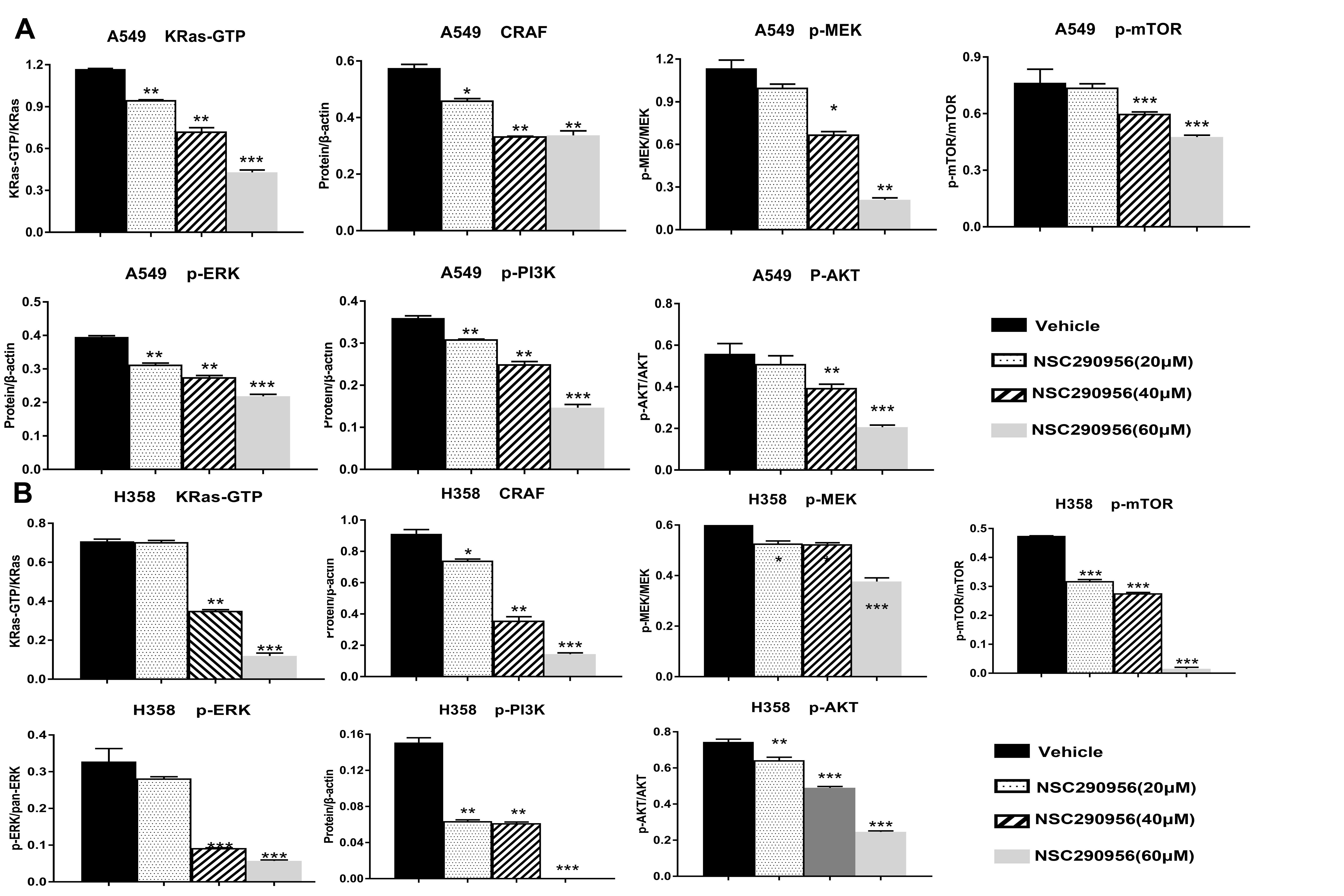


**Supplementary Figure 4. Related to Figure 5C**. (A) Immunoblots show shRNA-mediated knockdown of KRas in both A549 and H358 cell lines 12 hours after shRNA transfection. (B) The effect of knockdown on expression level of KRas was displayed by relative density analysis in both cell lines. (C) The effect of shRNA alone on cell viability was shown as the percentage of shNC-treated control. Significant differences from untreated control were indicated ^*^*p*< 0.05 and ^**^*p*< 0.01.

**
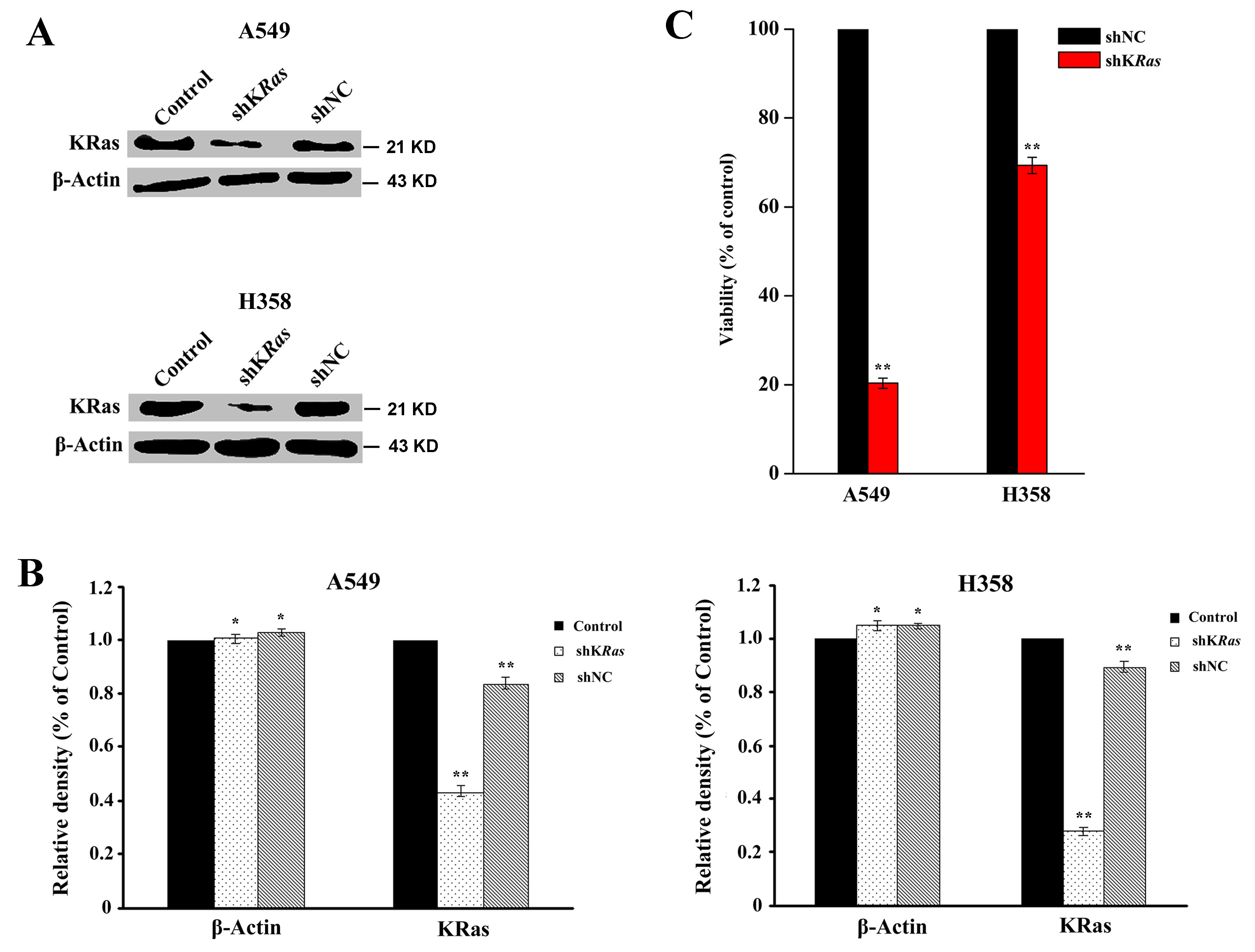
**

**Supplementary Figure 5. Related to Figure 5D**. Effect of NSC290956 on the expression levels of G2 phase-related proteins Cyclin B1 and CDK1 in both the cell lines. β-Actin was used as the internal control.

**
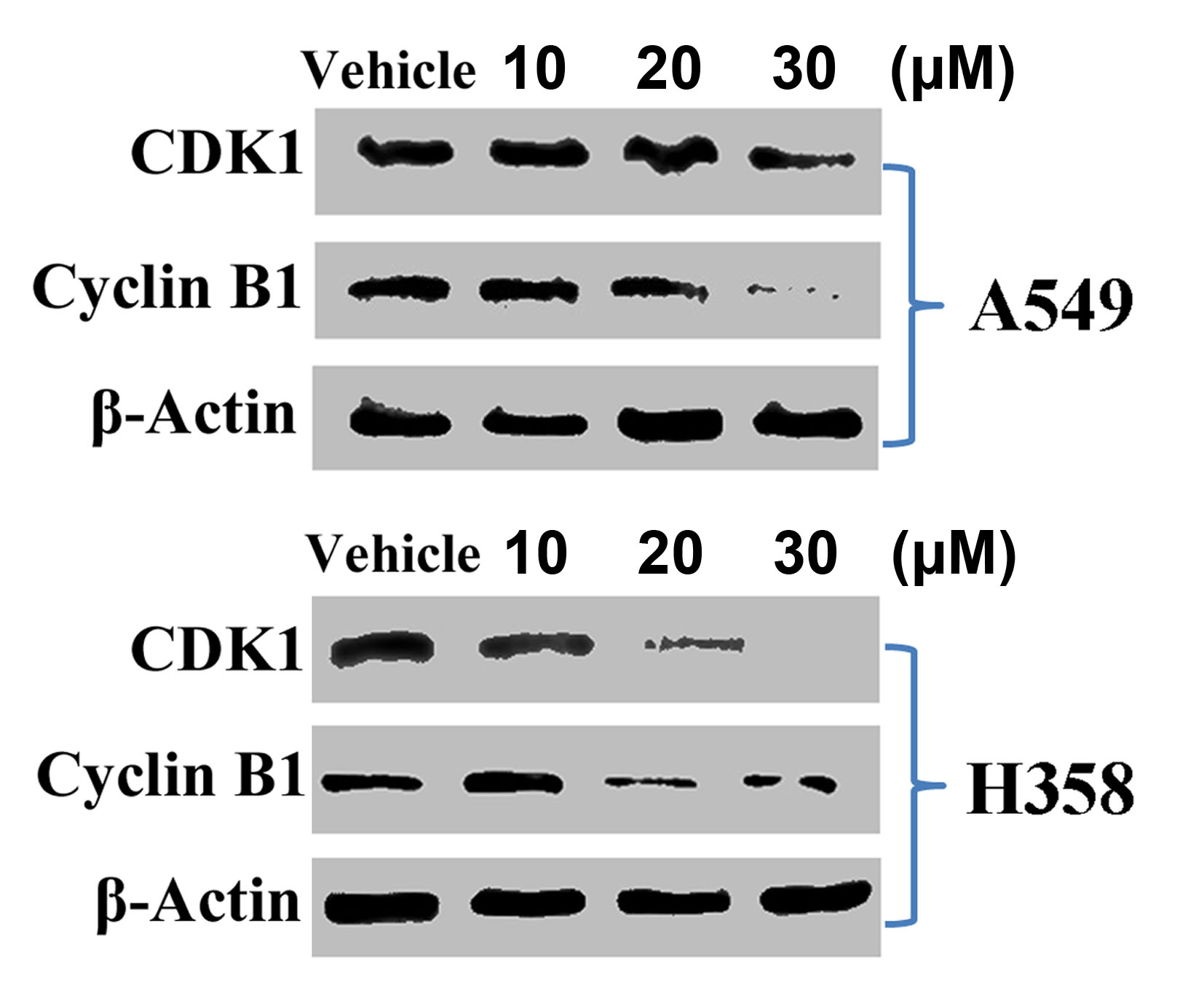
**

**
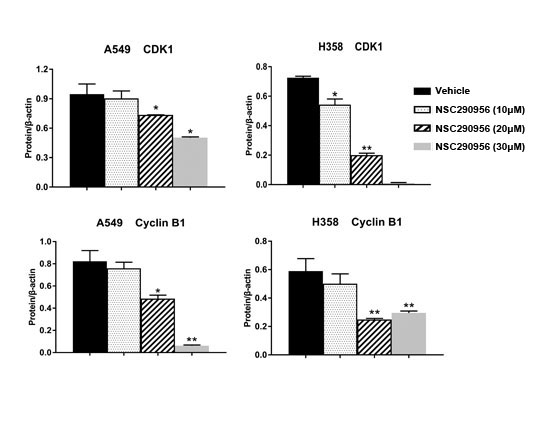
**

**Supplementary Figure 6. Related to Figure7.** (A) The total percentage of apoptotic cells (early apoptosis + late apoptosis) was analyzed by flow cytometry. Each value represents mean ± SD in three independent experiments. Significant differences from untreated control were indicated ^*^*p*< 0.05 and ^**^*p*< 0.01. (B) Nuclear morphological alteration and extent of apoptosis were examined using confocal laser scanning microscope. Representative results from three independent experiments are shown. The scale bar is 200 µm.

**
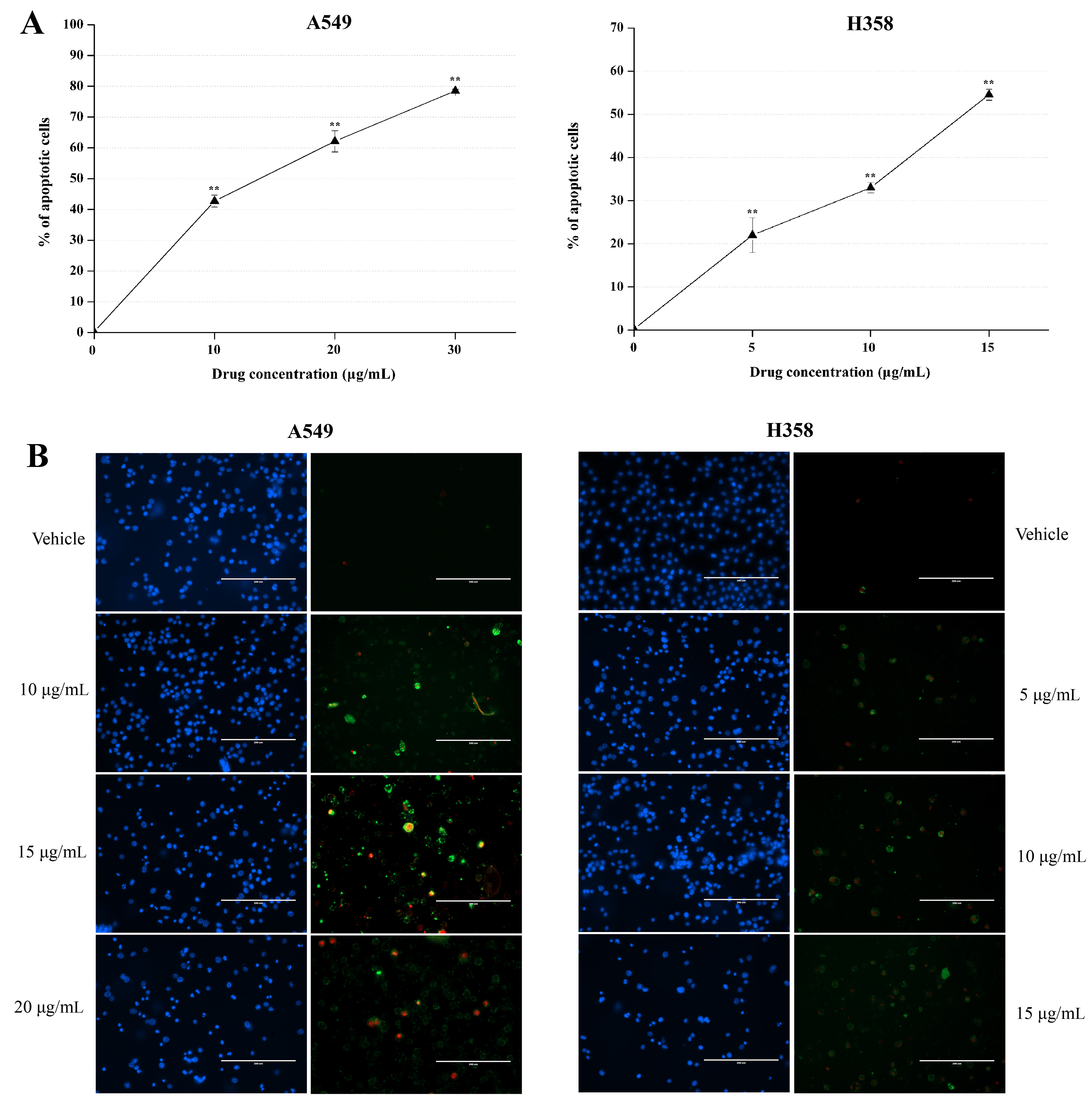
**

**Supplementary Figure 7. Related to Figure 7B,C.** NSC290956 regulated mitochondrial dysfunction for apoptosis in both A549 and H358 cells by WB analysis. Significant differences from untreated control were indicated ^*^*p*< 0.05, ^**^*p*< 0.01, and ^***^*p*< 0.001.


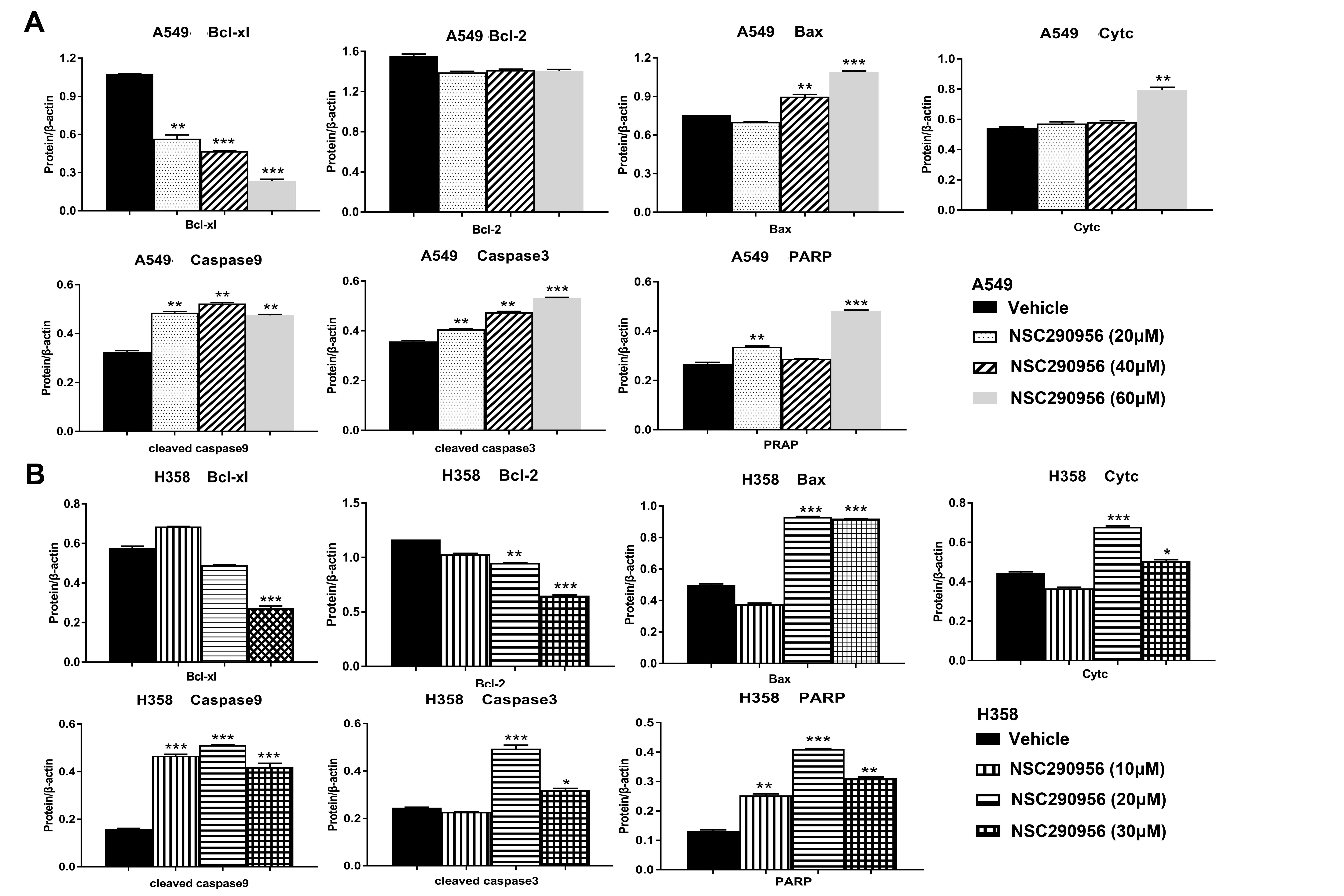


**Supplementary Figure 8. Related to Figure7**. The loss of mitochondrial transmembrane potential exhibited a dose-dependent increase after NSC290956 treatment. Both A549 and H358 cell lines treated at the indicated doses were subject to flow cytometry. Representative results from three independent experiments are shown.


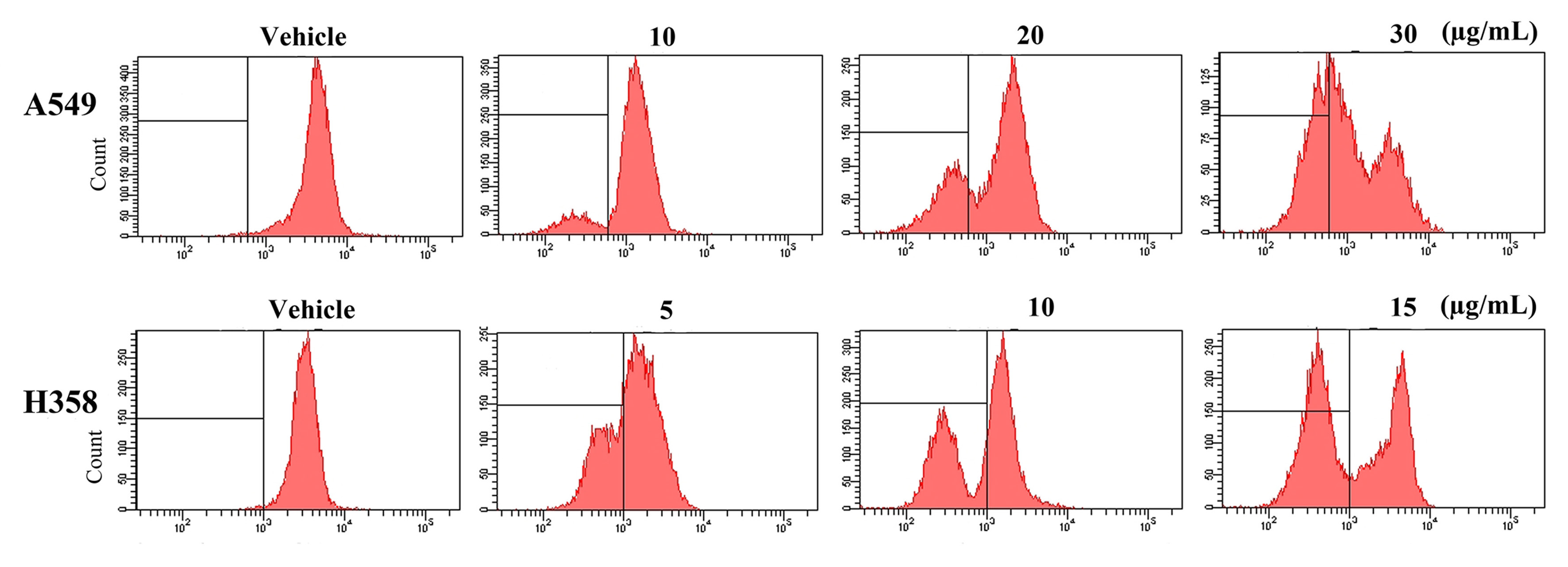


**Table S1 Details of hit candidates^*^**

| **Compound** | **NSC Number** | **CAS Number** | **Molecular Formula** | **Molecular**  **Weight (g/mol)** | **Binding Energy (kcal/mol)** | **ISR** |
| --- | --- | --- | --- | --- | --- | --- |
| 1 | 12363 | NF | C_26_H_14_N_2_O_3_ | 402 | -9.75 | 5.60 |
| 2 | 4292 | 5397-96-6 | C_19_H_12_N_2_O_3_S_2_ | 380 | -9.54 | 5.02 |
| 3 | 125034 | NF | C_18_H_12_N_2_O_2_S_2_ | 352 | -9.41 | 5.08 |
| 4 | 65828 | 6949-03-7 | C_22_H_17_N_3_O_4_S.Na | 442 | -9.35 | 4.15 |
| 5 | 7814 | 6283-28-9 | C_17_H_14_N_2_O_4_S.Na | 365 | -9.35 | 4.03 |
| 6 | 373058 | NF | C_21_H_16_N_2_O_2_ | 328 | -9.17 | 5.57 |
| 7 | 371684 | NF | C_24_H_18_N_4_O_2_ | 394 | -9.06 | 4.26 |
| 8 | 113909 | 19188-57-9 | C_18_H_19_FN_6_O_3_S.ClH | 455 | -9.04 | 4.09 |
| 9 | 133488 | 29519-87-7 | C_15_H_11_N_3_O_5_S | 345 | -8.73 | 5.94 |
| 10 | 117614 | 23113-01-1 | C_20_H_27_N_3_O_6_ | 405 | -8.65 | 4.94 |
| 11 | 168468 | NF | C_16_H_12_N_4_O_3_ | 308 | -8.63 | 4.36 |
| 12 | 168466 | 59034-56-9 | C_17_H_15_N_3_O | 277 | -8.53 | 4.45 |
| 13 | 656889 | NF | C_17_H_13_ClN_2_O_4_.Na | 368 | -8.52 | 6.42 |
| 14 | 299589 | 57808-66-9 | C_22_H_24_ClN_5_O_2_ | 426 | -8.37 | 7.01 |
| 15 | 94820 | 39673-98-8 | C_29_H_18_N_2_O_4_ | 458 | -8.36 | 5.44 |
| 16 | 98905 | 13896-97-4 | C_21_H_15_NOS | 329 | -8.34 | 4.07 |
| 17 | 310343 | NF | C_22_H_24_N_2_O_3_ | 364 | -8.26 | 4.23 |
| 18 | 145031 | NF | C_17_H_17_ClN_6_O_3_ | 389 | -8.30 | 5.09 |
| 19 | 48693 | NF | C_21_H_26_N_2_O_3_.2ClH | 427 | -8.29 | 5.20 |
| 20 | 290956 | 27007-85-8 | C_22_H_24_ClN_3_OS_2_.ClH | 482 | -8.10 | 5.54 |
| 21 | 79050 | 41193-15-1 | C_28_H_26_N_2_O_2_ | 423 | -8.17 | 5.08 |
| 22 | 646824 | NF | C_17_H_11_N_3_O_7_ | 369 | -8.05 | 5.02 |

**Continued Table S1 Details of hit candidates**

| **Compound** | **NSC Number** | **CAS Number** | **Molecular Formula** | **Molecular**  **Weight (g/mol)** | **Binding Energy (kcal/mol)** | **ISR** |
| --- | --- | --- | --- | --- | --- | --- |
| 23 | 48160 | 6640-90-0 | C_18_H_29_NO | 275 | -8.06 | 4.83 |
| 24 | 34240 | 6323-43-9 | C_20_H_28_N_2_S_2_ | 361 | -8.17 | 4.78 |
| 25 | 109131 | 21170-27-4 | C_27_H_34_N_4_O_2_S | 479 | -8.15 | 4.66 |
| 26 | 47938 | 6642-12-2 | C_22_H_19_NO_3_ | 345 | -8.05 | 4.44 |

^*^ indicates that all chemical data were acquired from the website: https://dtp.cancer.gov/dtpstandard/ChemData/index.jsp.

NF means the CAS number was not found fromthe website: https://dtp.cancer.gov/dtpstandard/ChemData/index.jsp.

**Table S2 Chemical names^*^**

| **NSC Number** | **Chemical Name** |
| --- | --- |
| 4292 | 1-(Naphthalen-2-yl)-2-(6-nitrobenzo[d]thiazol-2-ylthio)ethanone |
| 7814 | 2-Naphthalenesulfonic acid, 6-hydroxy-5-[(4-methylphenyl)azo]-, monosodium salt |
| 12363 | 1,2-bis(9-oxo-9H-fluoren-2-yl)diazene oxide |
| 34240 | N-((benzo[d]thiazol-2-ylthio)methyl)-N-cyclohexylcyclohexanamine |
| 47938 | 3-benzyl-3,4-dihydro-2H-benzo[e][1,3]oxazin-6-yl benzoate |
| 48160 | 4-tert-butyl-2-((cyclohexylamino)methyl)-6-methylphenol |
| 48693 | 2-(benzo[d]oxazol-3(2H)-ylmethyl)-5-((cyclohexylamino)methyl)benzene-1,4-diol, dihydrochloride |
| 65828 | 2-Naphthalenesulfonic acid, 8-amino-5-[(4’-hydroxy [1, 1’-biphenyl]-4-yl)azo]-, monosodium salt |
| 79050 | Naphtho[2,1-e:6,5-e’]bis[1,3]oxazine, 1,2,3,7,8,9-hexahydro-2,7-bis(phenylmethyl) |
| 94820 | 2,2’-(4,4’-methylenebis(4,1-phenylene))diisoindoline-1,3-dione |
| 98905 | 2-(phenanthridin-6-ylthio)-1-phenylethanone |
| 109131 | 10,13-dimethyl-17-(2-(6-(methylthio)-9H-purin-9-yl)acetyl)-6,7,8,9,10,11,12,13,14,15,16,17-dodecahydro-1H-cyclopenta[a]  phenanthren-3(2H)-one |
| 113909 | 4-(3-(4,6-diamino-2,2-dimethyl-1,3,5-triazin-1(2H)-yl)benzamido)benzene-1-sulfonyl fluoride hydrochloride |
| 117614 | 1-beta-D-Arabinoofuranosylcytosine 5’-adamantoate |
| 125034 | 2,2’-disulfanediyldiquinolin-8-ol |
| 133488 | 4-(2-(2-oxoindolin-3-ylidene)hydrazinylsulfonyl)benzoic acid |
| 145031 | N-(2-chloro-5-nitrobenzyl)-9-(tetrahydro-2H-pyran-2-yl)-9H-purin-6-amine |
| 168466 | 2-(1-(quinolin-2(1H)-ylidenehydrazono)ethyl)phenol |
| 168468 | 2-nitro-6-((quinolin-2(1H)-ylidenehydrazono)methyl)phenol |
| 290956 | 1-Thia-4,8-diazaspiro[4.5]decan-3-one, 8-[3-(2-chloro-10H-phenothiazin-10-yl)propyl]-, monohydrochloride |
| 299589 | 5-Chloro-1-[1-[3-(2-oxo-1-benzimidazolinyl)propyl]-4-piperidyl]-2-benzimidazolinone |
| 310343 | 4-Methoxy-N'-(3,3,6,8-tetramethyl-1-oxo-3,4-dihydronaphthalen-2(1H)-ylidene)benzohydrazide |

**Continued Table S2 Chemical names**

| **NSC Number** | **Chemical Name** |
| --- | --- |
| 371684 | 5,5'-bis(2-methyl-1H-indol-3-yl)-3,3'-biisoxazole |
| 373058 | 2-(3-Hydroxy-6-methylquinoxalin-2-yl)-1-(naphthalen-2-yl)ethanone |
| 646824 | 2-(benzo[d][1,3]dioxol-5-yl)-2-hydroxy-1-(3-hydroxy-6-nitroquinoxalin-2-yl)ethanone |
| 656889 | Propanoic acid, 2-[4-[(7-chloro-2-quinoxalinyl)oxy]phenoxy]-,sodium salt |

^*^ indicates that all chemical data were acquired from the website: https://dtp.cancer.gov/dtpstandard/ChemData/index.jsp.
